# Toward AI ecosystems for electrolyte and interface engineering in solid-state batteries

**DOI:** 10.1126/sciadv.aea0638

**Published:** 2025-11-26

**Authors:** Zhilong Wang, Wolfgang G. Zeier, Fengqi You

**Affiliations:** ^1^ Cornell University AI for Science Institute, Cornell University, Ithaca, NY 14853, USA.; ^2^ College of Engineering, Cornell University, Ithaca, NY 14853, USA.; ^3^ Cornell AI for Sustainability Initiative (CAISI), Cornell University, Ithaca, NY 14853, USA.; ^4^ Institute of Inorganic and Analytical Chemistry, University of Münster, Münster, Germany.; ^5^ Institute of Energy Materials and Devices, IMD-4 Helmholtz-Institute Münster, Forschungszentrum Jülich, Münster, Germany.

## Abstract

Solid-state batteries (SSBs) are pivotal for sustainable energy storage, delivering extended life span, low-temperature resilience, and enhanced safety. However, designing stable solid electrolytes and interfaces in SSBs remains a formidable challenge. As a disruptive catalyst for paradigm shifts spanning materials discovery and energy system redesign, artificial intelligence (AI) is unleashing unprecedented possibilities—could it be the key breakthrough for SSB innovation? Here, we critically review the progress of AI applications in electrolyte and interface engineering, covering key aspects such as stability, conductivity, mechanical properties, and interface resistance. This work emphasizes the integration of cutting-edge modeling strategies, including the materials’ screening pipelines, machine learning force fields, and generative models. Furthermore, we conduct an in-depth analysis of persistent challenges and propose a roadmap featuring multiscale modeling and multimodal models with physical constraints to build an intelligent ecosystem for SSB development. This review is expected to inspire interdisciplinary collaborations and drive forward energy materials design, ultimately accelerating the development of sustainable and cutting-edge battery technologies.

## Teaser

A critical review of AI applications in electrolyte and interface engineering for solid-state batteries is presented.

## INTRODUCTION

As the energy density of conventional liquid lithium-ion batteries (LIBs) approaches the theoretical limit, solid-state batteries (SSBs) using solid electrolytes (SEs) have emerged as a transformative next-generation energy storage technology, garnering notable attention for their unparalleled energy density and enhanced safety profiles (*1*, *2*). SSBs also play a crucial role in advancing sustainability and clean energy technologies by offering a safer, more efficient, and environmentally friendly energy storage solution (*3*, *4*). The deployment of SSBs contributes to lower carbon footprints and more sustainable energy storage and is expected to catalyze breakthroughs in strategic sectors such as electric vehicles and aerospace, thereby ushering in a new era of energy innovation (*5*, *6*). Recently, substantial advancements have been made in the performance of SSB technology (*7*–*9*). For instance, homogeneous cathodes composed entirely of Li_1.75_Ti_2_(Ge_0.25_P_0.75_S_3.8_Se_0.2_)_3_ have enabled room-temperature SSBs to achieve a cycle life exceeding 20,000 cycles at 2.5 C and a high energy density of 390 Wh/kg at 0.1 C (*2*, *10*). Organic-inorganic hybrid SEs with remarkable ionic conductivity at room temperature have also been designed for SSBs with superior rate capability and long-time cycling stability (*11*, *12*). Although theoretical mechanisms have been preliminarily explored, several challenges remain for SSB commercialization, particularly the intricate interplay between ionic transport, mechanical stability, and interfacial phenomena (*13*, *14*).

**Fig. 1.**
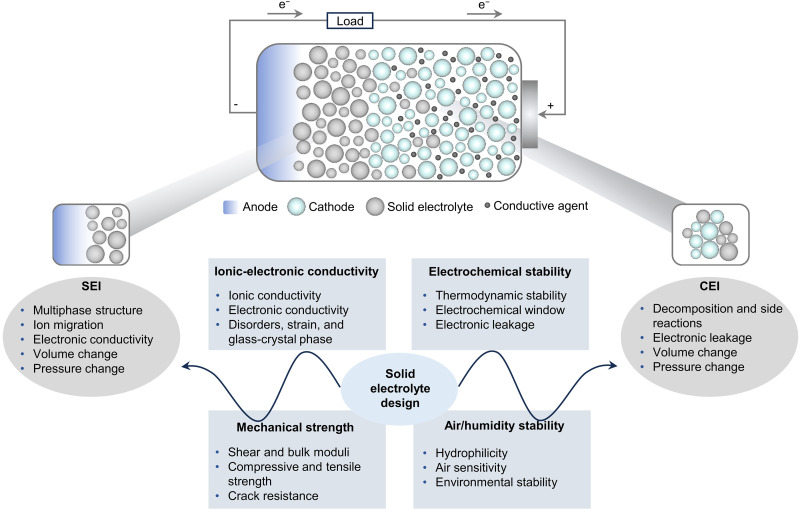
Key scientific goals and challenges for SE and SEI/CEI design. The factors that have to be considered for the SE design are demonstrated, including ionic-electronic conductivity, electrochemical stability, mechanical strength, and air/humidity stability, to address key scientific problems associated with SEI/CEI. This review aims to provide comprehensive AI-driven applications in SE design and SEI/CEI optimization. The anode, cathode, and SE are shown figuratively and do not represent their actual structures.

A critical challenge lies in understanding and optimizing Li^+^ transport in SE materials, where dynamic and static disorders of both anions and cations, along with strain effects, influence ionic conductivity. The presence of local distortions, correlated hopping mechanisms, and the competition between percolative and nonpercolative transport pathways complicates conventional transport models, necessitating a more refined understanding of coherence length and local defect chemistries. Furthermore, SEs must exhibit exceptional mechanical integrity to prevent crack propagation, stress accumulation, and interfacial delamination, all of which can lead to catastrophic failure during Li metal deposition and stripping (*15*, *16*). Beyond bulk transport, interfacial stability, particularly at the solid electrolyte interphase (SEI) (*17*–*20*) and cathode electrolyte interphase (CEI) (*21*, *22*) (Fig. 1), is the key for SSBs. The compositions, structures, and coupled ionic-electronic conductivities of SEI/CEI dictate the electrochemical kinetics of Li^+^ transport, yet their evolution under electrochemical cycling and mechanical stress requires systematic investigation (*23*, *24*). The electrochemical kinetics of Li^+^ transport through SEI/CEI layers remains insufficiently understood (*25*, *26*). The formation of space-charge layers, pressure-induced phase transformations, and interphase growth kinetics further complicate Li^+^ transport (*27*, *28*), leading to nonuniform current distributions and potential dendrite penetration pathways. In addition, volumetric fluctuations at the interfaces introduce further challenges, as local stress concentrations can induce mechanical failure or exacerbate interfacial instability.

In the era of artificial intelligence (AI), the emergence of large language models (LLMs) (*29*) and generative models (*30*) unlocks unprecedented opportunities for materials science and energy science. For instance, the materials generation platform MatterGen based on diffusion models can generate stable and diverse materials with mechanical, electronic, and magnetic properties (*31*), and the A-Lab, an autonomous laboratory for the solid-state synthesis, realized compounds including various oxides and phosphates (*32*). A computer-driven robotics laboratory also reported that their setup performed 224 reactions to make 35 inorganic compounds, including certain oxides commonly used in battery electrodes, offering an exciting platform for data-driven experimental science (*33*). The multimodal model and the semisupervised learning model also facilitate the discovery of SEs by reducing the reliance on large amounts of training data (*34*, *35*). For instance, combined graph convolutional neural networks (GCNNs) with quantum calculations, polymer electrolytes with suitable ionic liquids were efficiently found from more than 2200 candidates (*36*). Aiding with unsupervised learning, five SEs [M(dimethylformamide)_2_Ni(CN)_4_, M = Mn^2+^, Fe^2+^, Co^2+^, Ni^2+^, or Cu^2+^] were selected from 308 candidates, reducing experimental and computational costs (*37*). Via composition-structure bimodal learning and GCNN, six inorganic ion conductors (e.g., Li_6_Ti_2_S_6_ and Li_2_NbOF_5_) were identified with high ionic conductivity at 300 K (*35*). Although the complex compositions, phases, atomic arrangements (anion, cation, etc.), and dynamic/static disorders have not been fully captured, this progress provides inspirations on AI-driven advancements in key materials for SSBs, particularly for linking their electronic and ionic conductivity, mechanical properties, and other key characteristics to their performance (*38*–*40*).

AI approaches offer an efficient and low-cost way to discover high-conductivity SEs and unveil electrolyte-interface chemistries. Also, AI-driven simulations and automated experimentation enable precise control over interfacial stability, mitigating dendrite formation and interphase degradation. The ability to rapidly design materials, optimize processing conditions, and decode complex ion transport mechanisms enhances battery performance, longevity, and safety, driving the next generation of high-energy-density, durable, and commercially viable SSBs. More importantly, AI-enhanced SE and interfacial engineering minimizes harmful side reactions and prolongs battery life span, decreasing reliance on resource-intensive recycling processes and contributing to a cleaner energy ecosystem.

Recent retrospective reviews have summarized advancements in SSB development (table S1), highlighting the feasibility of designing SEs and optimizing SEI/CEI layers to achieve safe and energy-dense SSBs through experimental and computational approaches (*41*). These studies emphasize the importance of understanding ion transport mechanisms within SEs and across interfaces, particularly in addressing challenges such as low ionic conductivity and high interfacial resistance in inorganic SEs (*42*). Key design requirements for next-generation SEs have been outlined, focusing on chemical, geometric, mechanical, electrochemical, and interfacial transport properties essential for practical SSB applications. Furthermore, the integration of experimental findings with computational predictions has deepened the understanding of interfacial reactions, guiding future interface engineering efforts (*17*). In addition, the diversity of materials, research methodologies, and collaborative efforts has been identified as crucial for developing fast-conducting SEs and ensuring long-term SSB performance (*6*). Numerous reviews also provide theoretical progress updates on various SE types, including garnet-type (*43*) and NASICON-type (*44*) materials, alongside interface design strategies (*17*, *45*), offering valuable perspectives and actionable measures for future development. However, it can be observed that there are currently relatively few comprehensive discussions on AI-driven SE and interface design (table S1). This can be attributed to two key factors. First, the integration of experimental science and computational materials science is still insufficient, and AI, as an independent discipline, has yet to be fully integrated into SSB research, further exacerbating the scarcity of SE-related data. Second, compared to liquid LIBs, the experimental instruments and equipment required for SSB research are expensive, posing substantial challenges to commercialization and hindering rapid technological progress. Overall, computational and AI-based studies in SSB research are still in the preliminary stages.

Several critical knowledge gaps hinder the full potential of AI in optimizing SEs and SEI/CEI layers. On the materials science side, the multiscale nature of SEs, ranging from atomic-level ion migration to macroscopic mechanical stress distribution, remains poorly understood (*46*, *47*). The coupling effects of electron/ion transport properties, electrochemical, mechanical, thermal, and kinetic factors on interface stability are not quantitatively described, making it challenging for AI to accurately predict performance. On the AI-computational side, the lack of high-quality, standardized datasets limits the training and generalization capabilities of AI models, even though the active learning strategies have been used to alleviate the challenge of data scarcity in specific systems (*48*). Experimental data on ionic conductivity, interfacial stability, and mechanical properties are often sparse and inconsistent; the theoretical framework linking materials composition, interfacial behavior, and overall battery performance is incomplete. For instance, the kinetics and thermodynamics of SEI/CEI formation are not well defined, and the impact of interface modifications (e.g., coatings or buffer layers) on ion transport and impedance is unclear (*49*). Moreover, the feedback loop between AI predictions and experimental validation for SSBs is underdeveloped. The slow pace of experimental validation for the candidate materials and the absence of automated high-throughput materials characterization platforms hinders the iterative optimization of AI models. Addressing these gaps requires interdisciplinary efforts, combining advanced characterization techniques, multiscale simulations, and explainable AI frameworks to accelerate the development of high-performance SSBs.

**Fig. 2.**
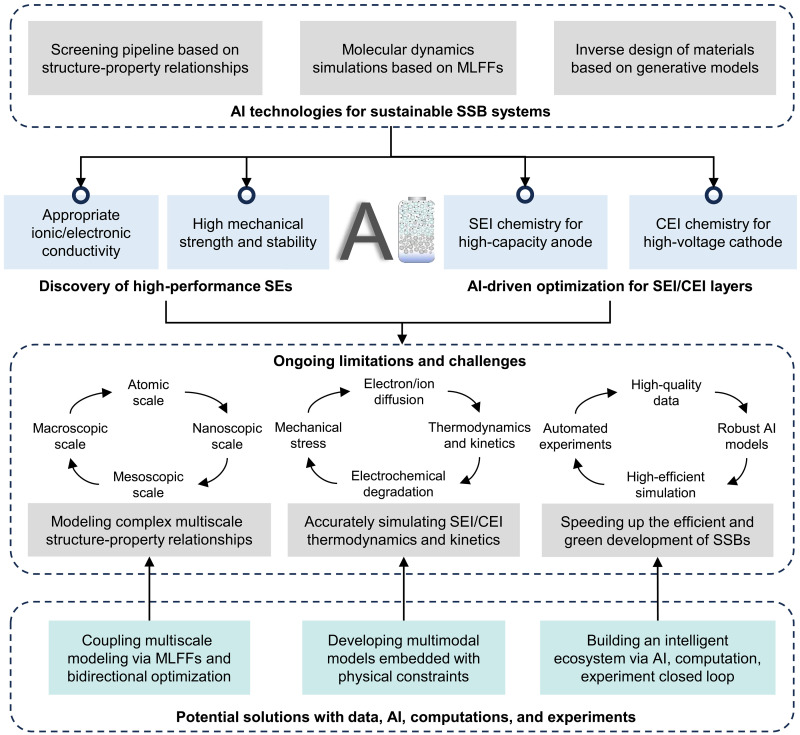
Overview of the review. First, three AI strategies for the SSB research are summarized. Next, advances in discovery of high-performance SEs with high ionic conductivity, as well as high mechanical strength and stability are reviewed and analyzed. Then, advances in SEI and CEI engineering are summarized and discussed. Last, three key ongoing limitations and corresponding potential solutions associated with SE and SEI/CEI engineering are proposed.

In this review, we focus on two critical aspects: electrolyte and interface design for SSBs, with a particular emphasis on the application of AI in their prediction and optimization, including (electro)chemical stability, ionic/electronic conductivity, mechanical properties, and interface resistance (Fig. 2). We systematically summarize recent advancements in the three most widely adopted approaches in SSB research, integrating state-of-the-art AI methodologies with materials science, chemical science, and energy science. Also, we critically analyze the strengths of these methods and propose potential directions for improvements. Last, we provide more insights into the remaining challenges for SSB development, and propose potential solutions and opportunities in the SE and SEI/CEI design, focusing on multiscale materials simulation, multimodal modeling, and intelligent platform establishment. The closed loop consisting of AI, computations, and experiments will hopefully create an intelligent ecosystem for the research and development (R&D) of SSBs, promote multifield cooperation, and accelerate the popularization of SSBs.

### AI strategies for electrolyte and interface design

**Fig. 3.**
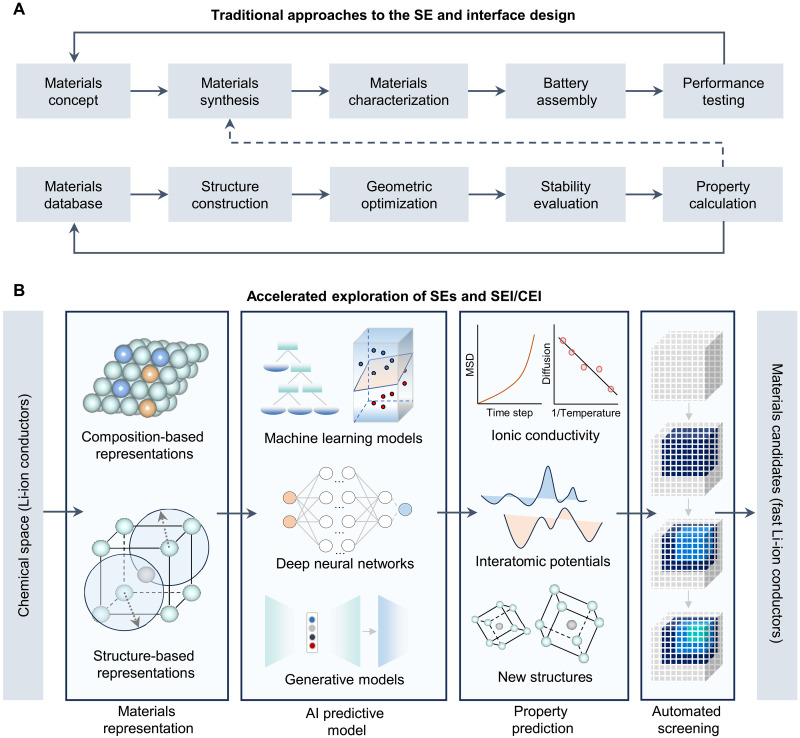
Strategies for SE and interface design. (**A**) Traditional approach to SE, including experimental and theoretical processes. Solid polylines denote the feedback of performance testing or property calculation, while the dashed polyline is the feedback of computational results to the experimental process. (**B**) Accelerated exploration of SEs and SEI/CEI layers using AI-driven methods. The process can be divided into four core parts: materials representation, AI predictive model, property prediction, and automated screening. Three AI-driven modes are summarized: establishing a screening pipeline based on structure-property relationships, performing MD simulations based on MLFFs, and designing materials based on generative models.

Traditional experimental and computational approaches typically rely on extensive trial-and-error processes, which often result in prolonged research cycles and substantial costs (Fig. 3A). AI has demonstrated its important role in both the discovery and design of SE and the investigation of ion transport and stability at SEI/CEI, by developing predictive surrogate models that learn structure-property or composition-performance relationships. These models are then embedded into an optimization framework to guide the search for optimal interfacial chemistries or formation protocols. Depending on the application, the problem may be treated as single-objective optimization or as multiobjective optimization, where a Pareto front is constructed to capture the trade-offs between competing objectives (e.g., maximizing conductivity while minimizing interfacial resistance)

$$\text{Single}-\text{objective}\;\left(\text{surrogate guided}\right):\left\{ \begin{array}{c} \underset{x\in \chi }{\text{min}}f\left(x\right)\\ s.t.g_{i}\left(x\right)\leq 0,i=1,2,\ldots ,m \end{array}\right.$$ (1)

$$\text{Multiobjective}\;\left(\text{Pareto}\right):\left\{ \begin{array}{c} \underset{x\in \chi }{\text{min}}\left[f_{1}\left(x\right),f_{2}\left(x\right),\ldots \right]\\ s.t.g_{i}\left(x\right)\leq 0,i=1,2,\ldots ,m \end{array}\right.$$ (2)

where *x* denotes decision variables in the feasible search space χ [e.g., interphase composition ratios (LiF fraction 0 to 1), formation protocol parameters (voltage 0 to 5 V, temperature 20 to 120°C, and rest time 0 to 24 hours), and additive concentration (0 to 10 wt %)], *f*(*x*) is the objective function [e.g., bandgap >1 eV, ionic conductivity ≥1 mS cm^−1^, electrochemical stability window (≥4.5 V), and Li plating uniformity index], and *g_i_*(*x*) gives physical or practical constraints (e.g., chemical compatibility ≤ threshold reaction energy, cost ≤ target). Scalarization methods such as the weighted sum approach or the ε-constraint method can be used to convert the multiobjective problem into a single-objective form for efficient search.

One may predict interfacial stability, decomposition energy, or Li^+^ transport resistance as a function of composition (*50*), and use Bayesian optimization (BO) to navigate the design space. For instance, by defining the design variables Li, cation, and anion site occupancies within the structures, BO coupled with a Gaussian process surrogate model was able to identify promising SEs with bandgap >1 eV and energy above hull <50 meV. Only 14 iterations were required to locate candidates within a search space of 4183 materials (*51*), demonstrating the efficiency of BO in navigating large compositional-structural design space.

The AI-prediction results can be integrated with experimental techniques such as x-ray photoelectron spectroscopy (XPS) technology and electrochemical impedance spectroscopy (EIS), as well as with density functional theory (DFT) methods like ab initio molecular dynamics (AIMD) simulations (*52*). This integration not only accelerates both experimental and theoretical research but also substantially reduces costs associated with trial-and-error processes. In this section, drawing on these studies, the applications of ML and DL are summarized and categorized into three primary modes (Fig. 3B): establishing screening pipeline based on structure-property relationships, performing MD simulations based on machine learning force fields (MLFFs) (*53*–*55*), and designing materials based on generative models. This induction aims to serve as a reference for more effective application of these methods and the design of more advanced techniques.

#### Establishing screening pipeline based on structure-property relationships

Accelerating the discovery and design of key materials (i.e., SEs and coating materials) is critical to advancing the development of SSBs. Establishing robust structure-property relationships that bridge materials structures and chemical compositions to their intrinsic properties remains the dominant paradigm for the accelerated discovery of materials. A predictive pipeline built upon this framework enables efficient screening and optimization of candidate materials. The performance and generalizability of predictive models are highly dependent on the quality, diversity, and representation of training data. Proper data analysis and feature engineering play a pivotal role in ensuring that the model captures meaningful chemical and physical trends rather than spurious correlations.

Comprehensive materials databases, such as the Inorganic Crystal Structure Database (ICSD) (*56*), Materials Project (MP) (*57*), Novel Materials Discovery (*58*), Automatic Flow for Materials Discovery (*59*), and Open Quantum Materials Database (*60*), house millions of structural entries alongside experimentally measured and DFT-computed properties, including formation energy, bandgap, electronic structure, and elastic properties. These extensive datasets serve as the foundation for constructing high-precision ML or DL models for property prediction. A critical aspect of this process lies in transforming structural and compositional information into effective “descriptors” or “fingerprints,” which fundamentally dictate the predictive performance and generalizability of the models. To facilitate this, numerous advanced materials informatics toolkits, such as DeepChem (*61*), Matminer (*62*), Pymatgen (*63*), AlphaMat (*64*), and MAST-ML (*65*), provide robust functionalities for generating and analyzing a diverse array of materials descriptors, thereby enhancing the efficiency and accuracy of computational materials design. In the practical application, rigorous data curation, outlier detection, and domain-specific feature extraction, such as symmetry-aware descriptors or physically motivated embeddings, are essential preprocessing steps. The strategies not only improve model robustness but also enhance interpretability and reduce error risks in SE and SEI/CEI modeling tasks. Additional practical guardrails should be incorporated, including data leakage checks to ensure no near-duplicate structures appear across training/validation/test splits, temperature normalization for ionic conductivity, measurements to allow fair comparisons, and the creation of dataset cards containing licenses and provenance information.

Several studies have explored the prediction of SE and SEI/CEI properties, to identify high-performance materials within the vast and uncharted unknown chemical space. For instance, the electronic conductivity of garnet-type SEs was predicted based on their chemical compositions, enabling the screening of electronically insulating garnet-type SEs from more than 20,000 candidate compositions generated through elemental substitution (*66*). In addition, a hybrid fingerprint incorporating structural, compositional, and site information was developed to represent Li^+^ conductors, facilitating the prediction of ionic conductivity at an experimental/AIMD level (*67*). By leveraging predictive modeling of materials properties, ideal SEs and coating materials can be systematically screened from vast chemical spaces, achieving high-throughput materials discovery through the seamless integration of theoretical verification with experimental implementation.

#### Performing MD simulations based on MLFFs

Unlike conventional approaches that establish direct mapping relationships between materials and their properties, the construction of MLFFs, also known as ML potentials (MLPs), represents a cutting-edge technology for delving into the thermodynamic mechanisms of materials in unprecedented detail. MLFFs predict potential energy *E* of a system as a function of atomic coordinates {*r_i_*} and decompose *E* into atomic contributions to improve interpretability and training efficiency

$$E=\sum_{i=1}^{N}E_{i}$$ (3)

$$E_{i}=M\left(\{r_{\mathrm{ij}}\},j\in K\left(i\right)\right)$$ (4)

where *E_i_* is the local contribution of atom *i*, depending on the relative coordinates *r_ij_* = *r_i_* – *r_j_* of its neighbors *K*(*i*). *M* is the ML model, such as graph neural networks (GNNs) and LLMs. GNNs let atoms be graph nodes with hidden states $h_{i}^{t}$ (e.g., atomic number, oxidation state, and electronegativity), and edges carry geometric features $e_{\mathrm{ij}}$ (e.g., radial and angular terms). A single message-passing layer with learnable function $\phi_{m}$ is

$$m_{\mathrm{ij}}^{\left(t\right)}=\phi_{m}\left(h_{i}^{t},h_{j}^{t},e_{\mathrm{ij}}\right),{\tilde{m}}_{i}^{t}=\sum_{j\in K\left(i\right)}m_{\mathrm{ij}}^{\left(t\right)}$$ (5)

When using LLMs, *M* can be obtained from a transformer attention layer over neighboring atoms

$$a_{\mathrm{ij}}=\frac{\text{exp}\left(\frac{q_{i}^{⊤}k_{j}}{\sqrt{d}}\right)}{\sum_{j\in K\left(i\right)}\text{exp}\left(\frac{q_{i}^{⊤}k_{j}}{\sqrt{d}}\right)},h_{i}^{\prime }=\sum_{j\in K\left(i\right)}a_{\mathrm{ij}}v_{j}$$ (6)

where $q_{i}=W_{Q}h_{i}$ (query), $k_{j}=W_{k}h_{j}$ (key), $v_{j}=W_{V}h_{j}$ (value), and *d* is the key dimension. The tokenization for LLMs can be formulas (e.g., Li_10_GeP_2_S_12_), Wyckoff/lattice tokens, fractional coordinates, or space group embeddings.

Forces $F_{i}$ are derived from their negative gradient, ensuring energy conservation.

$$F_{i}=-\nabla_{r_{i}}E$$ (7)

To enhance generalization, loss functions often include physics-driven regularization terms. For example, a joint loss for energy and forces

$$L=\lambda_{E}{\left\|E_{\mathrm{ML}}-E_{\mathrm{QM}}\right\|}^{2}+\lambda_{F}\sum_{i}{\left\|F_{i}^{\mathrm{ML}},-,F_{i}^{\mathrm{QM}}\right\|}^{2}$$ (8)

where $E_{\mathrm{QM}}$ and $F_{i}^{\mathrm{QM}}$ are reference values from quantum mechanical (QM) calculations, and $\lambda_{E}$ and $\lambda_{F}$ are hyperparameters balancing energy and force accuracy.

Leveraging conformational data derived from AIMD simulations, alongside energy and atomic force data obtained through DFT calculations, MLFFs can be developed with minimal error margins. These trained MLFFs effectively supplant the need for DFT calculations within AIMD, accelerating MD simulations. For instance, typical MLFF-based MD simulations can achieve speedups ranging from 10^3^ to 10^6^ compared to DFT with generalized gradient approximation functional, depending on the system size and the specific ML model used (*68*, *69*). This drastic improvement in efficiency allows for longer timescales and larger system sizes to be accessed, making it feasible to investigate rare events, defect dynamics that would otherwise be prohibitively expensive with DFT (see table S2). Furthermore, by accurately simulating long-range interactions, MLFFs facilitate the expansion of simulation systems from the atomic to the microscopic scale, enabling the exploration of dynamic mechanisms across various scales. Consequently, MLFFs are poised to revolutionize dynamic simulations by enhancing both temporal and spatial scalability. The forefront of MLFF construction toolkits tailored to diverse materials systems are provided in table S2.

MD simulations leveraging MLFFs enable highly precise and computationally efficient investigations into ion transport mechanisms, interfacial stability, and atomic-scale dynamic processes. These advanced simulations transcend the constraints of conventional force fields by adeptly capturing intricate interactions and long-range effects, thereby offering profound insights into materials behavior. For instance, the innovative concept of density of atomistic states (DOAS) was introduced with the aid of MLFFs, enabling the quantitative analysis, elucidation, and characterization of the energetics across a vast array of atomic states with disordered configurations in frustrated materials (*70*). The DOAS was further applied in guiding the rational design of fast-ion conductors. In another notable application, the ion diffusion mechanism in Li_6_PS_5_Cl at 300 K was meticulously examined through large-scale (6500 atoms), long-term (25 ns) MD simulations empowered by MLFFs (*71*). These simulations revealed that Li^+^ conductivity reaches its zenith when Cl^−^ occupies 25% of the 4c sites rather than at 50% where the disorder is at its peak. The critical factors influencing Li^+^ transport, as identified by MLFFs, are pivotal in optimizing SEs and SEI/CEI layers. Despite this, there is still a persistent gap between the experimental results and MD simulations that urgently needs to be addressed. This discrepancy arises from various factors, including limitations in the accuracy of force fields used in MD simulations, the challenges of replicating experimental conditions in silico, and the inherent complexity of disordered structural systems that often defy simplified computational models.

#### Designing materials based on generative models

Another mode for designing SEs and optimizing SEI/CEI involves leveraging generative models to generate materials with tailored properties, an inverse design strategy that maps desired materials properties back to viable structural compositions (*72*). Generative AI (GenAI) models, including multimodal LLMs (*73*) and agent technologies (*74*), offer unprecedented opportunities to explore unknown materials structures.

Although the application of generative models in molecular and materials science remains in its nascent stages, substantial progress has been made (table S3). For instance, molecular-level generative models, such as PocketFlow, a data knowledge–reinforced model (*30*), G-SchNet, a graph-based deep generative model for 3D molecular structures (*75*), and GT4SD, a general-purpose generative toolkit (*76*), focus on generating valid and diverse organic molecules for drug discovery and materials applications. For inorganic crystalline materials, models such as the physics-guided crystal generative model (PGCGM) (*77*), crystal diffusion variational autoencoder (*78*), CrystalGAN (*79*), UniMat (*80*), and score matching with Langevin dynamics (SMLD) (*81*) can generate structures with desired formation energies and high stabilities. More recently, diffusion-based models for crystalline materials (MatterGen) (*31*) and the Simplified Line-Input Crystal-Encoding System (*82*) have shown superior generation quality and property conditioning, especially for complex materials. Physics-integrated models like PGCGM and SMLD explicitly incorporate physical priors or energy constraints to ensure thermodynamic plausibility, while other conditional generation capabilities have become increasingly important, enabling inverse design targeting specific properties. These advancements suggest that generative models could revolutionize materials discovery, particularly in the search for unexplored crystal structures and chemical compositions for the high-performance SEs and coating materials.

Notably, GenAI has already been applied to the design of polymer electrolytes. For instance, by using pretrained transformer (GPT)-based and diffusion-based models trained on a dataset of 6024 amorphous polymer electrolytes, researchers have successfully captured the complex “language” of polymer electrolytes, enabling the generation of promising candidates with superior ionic conductivity (*83*). In addition, a conditional generative model based on the miniGPT architecture has been introduced to generate polymer structures (*84*). The efficacy of this framework was demonstrated by its identification of 14 unique polymer repeating units exhibiting computed ionic conductivities surpassing that of polyethylene oxide, highlighting the transformative potential of GenAI in electrolyte design. GenAI models have not been widely applied in SSB research due to the benchmark and data limitations. Nevertheless, MatterGen demonstrates the capability to generate crystalline materials with bandgap >3.0 eV (*31*), making it a promising tool for electronically insulating SE discovery. As comprehensive datasets on key SE properties (e.g., ionic conductivity and mechanical strength) continue to accumulate, such models may play an increasingly important role in generating high-performance SEs. For the SEI/CEI design, where the focus lies on the generation and evolution of interfacial structures, diffusion models enhanced with physics priors, such as atomic arrangements and charge balance, are particularly promising for inverse design. These models are well suited to capture the critical influence of physical constraints and local bonding environments, thereby enabling more reliable and interpretable generation of interfacial chemistries in SSBs.

To bridge the discussion from AI strategies to their practical applications in SE and interface design, we emphasize how screening pipelines, MLFFs, and generative models collectively contribute to the optimization process. Screening pipelines enable efficient candidate selection, MLFFs provide accurate atomistic simulations, and generative models facilitate the exploration of structures. These AI-driven approaches not only accelerate the identification of promising materials but also enhance our understanding of interfacial phenomena, setting the stage for their targeted application in SSB development.

### Discovery of high-performance SEs

#### SEs with high ionic conductivity

The ionic conductivity of SEs plays a pivotal role in advancing battery technology, as it directly influences the efficiency, energy density, cycle life, and safety of SSBs. Accurate and efficient prediction of ionic conductivity is essential for accelerating the development of materials, reducing experimental time, and minimizing costs (*85*–*87*). By establishing robust prediction and screening methods, researchers can identify promising materials and allocate resources more effectively, thereby enhancing R&D efficiency. This section highlights recent advancements in leveraging cutting-edge AI technologies to predict ionic conductivity and discover fast-ion conductors.

**Fig. 4.**
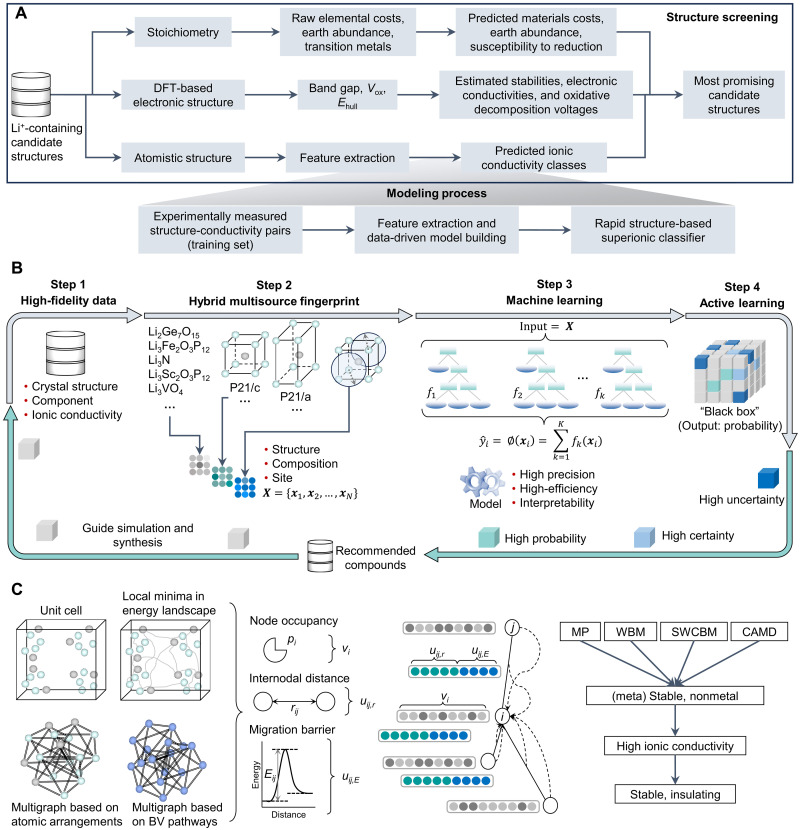
Mainstream methods for discovering fast-ion conductors. (**A**) Data-driven classification model using an LR algorithm for predicting ionic conductivity (*88*). Elemental costs, earth abundance, and environmental factors of raw materials were taken into account in the screening pipeline. Also, for the SE application, electronic conductivity was used as a screening indicator. (**B**) IonML platform for the fast-ion conductor discovery and design (*67*). A hybrid multisource fingerprint was integrated into the IonML, which describes the structural, compositional, and site information of fast-ion conductors. The active learning module can be used as an optimization tool to direct the design of chemical compositions for fast-ion conductors. (**C**) Predicting the ionic conductivity based on a combination of the bond-valence (BV) method and graph convolutional network (GCN) (*91*). Each BV node is associated with an occupancy *p_i_*, and each edge is associated with a distance *r_ij_* and an energy barrier *E_ij_*. The GCN is used to predict ionic conductivity from quantities derived from the BV energy landscape. The right part shows the workflow for high-throughput screening from the MP, WBM, SWCBM, and CAMD databases (~50,000 Li-containing compounds).

As early as 2016, considering factors such as structural and chemical stability, electronic insulation, and cost-effectiveness, a comprehensive library of 12,831 Li^+^-containing crystalline materials was constructed (*88*). Leveraging this dataset, researchers developed a data-driven classification model for ionic conductivity (threshold = 10^−4^ S/cm) using a logistic regression (LR) algorithm, chosen for its efficiency and ease of deployment (Fig. 4A). This rigorous selection process narrowed the candidate materials from 12,831 to just 21 promising structures, some of which have since been experimentally validated as viable SEs. Furthermore, the LR model revealed that simplistic atomic descriptors, such as volume per atom and Li bond ionicity, were insufficient predictors of ionic conductivity, underscoring the need for more sophisticated feature engineering in predictive modeling. Building upon this framework, the researchers conducted a targeted search for experimentally synthesized and characterized candidate materials, subsequently calculating ionic conductivity through AIMD simulations (*89*). Compared to an unguided, random exploration of the chemical space, the ML-driven approach demonstrated a 2.7-fold higher likelihood of identifying fast Li^+^ conductors. This underscores the transformative potential of ML in accelerating the discovery of high-performance SEs. In addition, these candidates were predicted to exhibit low electronic conductivity, exceptional oxidation resistance, and high thermodynamic stability, collectively positioning them as highly promising SEs.

For the prediction of ionic conductivity, modeling approaches leveraging limited high-fidelity experimental data remain the predominant method. Such frameworks not only ensure predictive accuracy but also often retain chemical interpretability, enabling the quantification of how chemical composition and the local Li^+^ environment influence Li^+^ migration dynamics, even when the training dataset fails to comprehensively span the entire chemical space. Recently, building on experimental datasets, researchers introduced a physically inspired ML platform, IonML (Fig. 4B), which uses hybrid multisource fingerprint (HMFP) descriptors integrating structural, compositional, and site features of Li^+^ conductors (*67*). This platform achieved a remarkable precision of 90.4% in classifying fast-ion conductors. To further harness the chemical insights derived from the model, they incorporated active learning for the targeted design of fast-ion conductors based on HMFP descriptors. This approach led to the identification of 126 fast-ion conductors with high ionic conductivity from a pool of more than 144,000 compounds, and eight structures that were not in the training dataset have been experimentally confirmed. By shortening the trial-and-error cycle and reducing associated costs, IonML addresses a critical gap in the design of fast-ion conductors.

While manually extracting features from a limited set of known materials can enhance the physical interpretability of descriptors, such an approach often fails to capture the nuanced structural features essential for accurate predictions. The advent of GNNs has revolutionized the ability to learn both the local chemical environment (LCE) and global structural information in crystalline materials. A high-throughput screening strategy integrating the bond-valence (BV) method (*90*) and graph convolutional networks (GCNs) has been introduced to identify promising SEs (Fig. 4C) (*91*). Notably, it reveals that the BV energy barrier between Li^+^ sites serves as the most critical input for GCN training, followed by the path length between nodes and the initial node occupancy. These findings highlight key priorities for future ionic conductivity modeling, including improving the fidelity of energy barrier calculations and integrating additional descriptors such as local transition rates. Expanding on the capabilities of GCNs, a platform, AI-IMAE, has been developed to predict the ion migration activation energy (*E*_a_) of both cationic [Li^+^, Na^+^, Ag^+^, Al^3+^, Mg^2+^, Zn^2+^, and Cu^(2)+^] and anionic (F^−^ and O^2−^) conductors (*92*), using the high-throughput computational database (*93*). With the continuous availability of high-precision training datasets, ranging from the AIMD level to the experimental level, the AI-IMAE platform enables users to train customized models and apply transfer learning (TL) techniques to adapt existing models to materials systems, ensuring the platform remains dynamically responsive to evolving research demands.

**Fig. 5.**
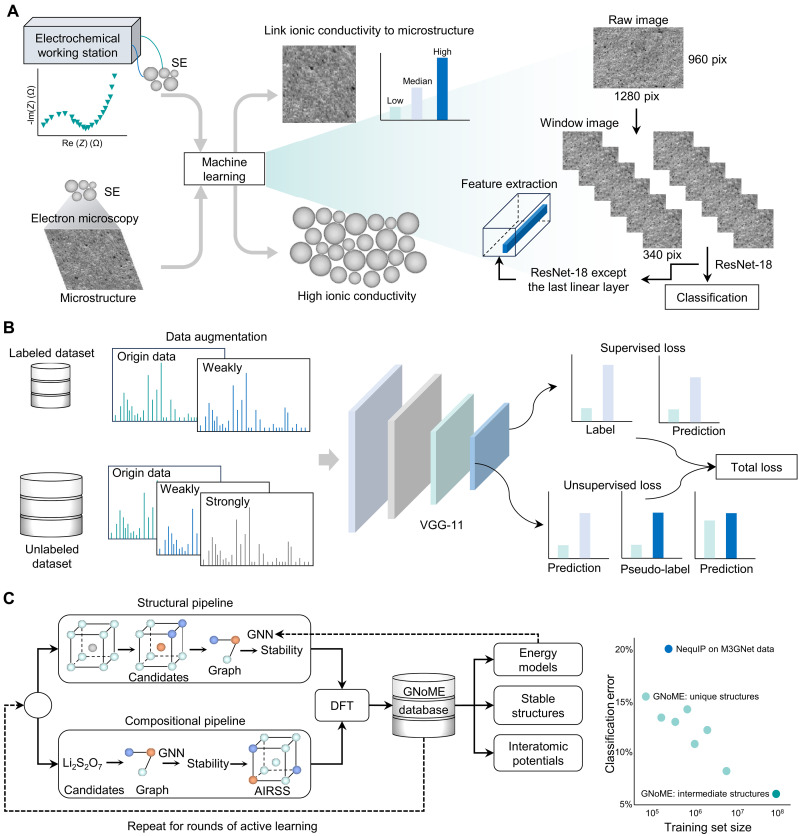
Discovery of fast-ion conductors using microstructures, x-ray diffraction, and machine learning interatomic potentials. (**A**) Microstructure-property relationship in solid electrolyte through the ResNet-18 model. The SE pellets (LLZTO) were prepared, and their ionic conductivities were measured (*94*). Scanning electron microscopy (SEM) was used to reveal the microstructure of the solid electrolyte pellets. The ionic conductivities of solid electrolyte pellets were divided into three levels: low, median, and high. (**B**) Semisupervised learning framework for predicting the ionic conductivity (*96*). Labeled data were derived from experimental results, while the unlabeled data were sourced from the ICSD (*56*). (**C**) Scheme of the GNoME platform (*39*). Massive structures were collected from both the structural and compositional pipelines to form the training dataset for the GNN model. An active learning strategy was used to enhance model accuracy and broader data coverage. The outputs of GNoME can be summarized as three parts: energy models, stable structures, and interatomic potentials. These interatomic potentials were then used to accelerate the AIMD simulations for discovering fast-ion conductors. With the same training data size, GNoME demonstrates a very low classification error in identifying fast-ion conductors. When intermediate structures are included in the training data, the classification error is reduced to ~5%.

In addition to these methods that establish relationships between the structure and ionic conductivity, several unique modeling approaches have emerged. For example, an algorithmic framework has been developed to elucidate the influence of a material’s microstructure on its performance by correlating microscopic morphological images with ionic conductivity (Fig. 5A) (*94*). Using a garnet-type Li_6.4_La_3_Zr_1.4_Ta_0.6_O_12_ (LLZTO) SE as a case study, the micromorphology was quantified by extracting certain physical parameters from images. Specifically, a bag-of-words model was constructed on the deep network features to summarize typical microstructures associated with varying properties and represent micrographs as meaningful vectors. These vectors were subsequently used to train a decision tree model, achieving a classification accuracy exceeding 90%, thereby successfully capturing the relationship between microstructure and ionic conductivity. This approach identifies key microstructural features of Li^+^ conductors with high ionic conductivity, offering valuable guidance for synthesizing highly conductive SEs.

Beyond microstructure, x-ray diffraction (XRD) patterns of crystalline materials have also been linked to ionic conductivity. Zhang *et al.* (*95*) pioneered the use of modified XRD, where the anionic lattice was set based on the S anion and subsequently scaled to the same atomistic volume, as a feature to cluster 28 representative anionic structures, uncovering common characteristics of fast Li^+^ conductors. Their unsupervised learning scheme found 16 unexplored fast Li^+^ conductors with conductivities ranging from 10^−4^ to 10^−1^ S/cm. While this method narrows the materials screening scope, it lacks direct structure-property relationships. To address this limitation, Wan *et al.* (*96*) introduced XRDMatch, a semisupervised learning framework, to discover fast-ion conductors (Fig. 5B). XRDMatch leverages a large volume of unlabeled data from the ICSD alongside a small set of labeled experimental data, overcoming data scarcity while ensuring accuracy and robustness. The model’s reliability was confirmed by successfully identifying the fast-ion conductor Li_6_AsSe_5_I, demonstrating its efficacy in accelerating materials discovery. Furthermore, the introduction of XRD descriptors eliminates the need for prior structural elucidation, streamlining model integration with experimental workflows and expediting the identification of materials. A summary of predictive models for discovering SEs with high ionic conductivity (*97*–*99*) is shown in table S4.

It is worth noting that the lack of structural data with crystallographic information files is a problem, and EIS measurements have combined contributions of grain boundary (GB) and in grain processes, resulting in the uncertainty of ionic conductivity. We emphasize that modeling and prediction using activation energy instead of ionic conductivity may be more accurate and reliable, as the temperature dependence is less sensitive to the absolute value of the ionic conductivity.

Another promising approach to accelerating the discovery of fast-ion conductors involves leveraging MLFFs with exceptionally low energy and atomic force errors, effectively replacing costly DFT calculations in AIMD simulations. Researchers then introduced GNoME (Fig. 5C), demonstrating that large-scale trained GNNs can achieve unprecedented levels of generalization, enhancing materials discovery efficiency by an order of magnitude (*39*). Building on 48,000 stable crystals identified in prior research, this approach enabled the discovery of 2.2 million structures below the current convex hull (*E*_hull_), many of which defy conventional chemical intuition. The GNoME potentials exhibit remarkable robustness in out-of-distribution, zero-shot settings and generalize effectively to high temperatures, making them a powerful tool for the high-throughput discovery of SEs, as illustrated in Fig. 5C.

Furthermore, MD simulations are indispensable for elucidating Li^+^ transport mechanisms in existing ionic conductors. Traditional approaches, which infer room-temperature ion diffusion properties from high-temperature (>600 K) AIMD simulations via the Arrhenius assumption, often introduce deviations (*66*, *100*). In contrast, ultralong MD simulations based on MLFFs provide a more accurate and efficient means of exploring ion diffusion events at low temperatures while maintaining DFT-level precision. For instance, low-temperature MLFF simulations revealed the nonlinear Arrhenius behavior of Li^+^ in Li_3_ErCl_6_, which has an ionic conductivity of 0.05 to 0.3 mS/cm (*101*), explaining why traditional AIMD simulations overestimate its ionic conductivity (*68*). A 1-μs MLFF simulation captured the multianion rotation events in Li_7_P_3_S_11_ at room temperature, highlighting rotational motion in four [PS_4_]^3−^ tetrahedra within the long-chain [P_2_S_7_]^4−^ group, while isolated [PS_4_]^3−^ groups remained static (*102*). These ultralong MLFF simulations also demonstrated that the paddle-wheel effect is absent in crystalline Li_6_PS_5_Cl at room temperature, with rotating [PS_4_]^3−^ polyanion groups exerting a slight negative influence on overall Li^+^ diffusion (*103*). Such insights deepen our understanding of the relationship between polyanion rotation and cation diffusion in ionic conductors, providing critical physical knowledge for designing ML models and developing more accurate force fields.

While AI-driven approaches have made strides in accelerating the discovery and optimization of SEs, several fundamental challenges remain. The interplay between static and dynamic disorder, stemming from factors such as anion and cation sublattice disorder (*104*–*106*), and strain effects (*107*), poses a barrier to accurate predictions. Furthermore, the coherence length, the spatial scale over which ion transport remains correlated, varies drastically across different SE types (*108*, *109*), particularly in systems exhibiting mixed glass-crystal phases or interfacial nanostructuring (*110*). Capturing these complex multiscale transport mechanisms requires AI models that go beyond conventional structure-property relationships, integrating physics-informed descriptors, real-time ionic dynamics, and adaptive learning frameworks capable of handling nonequilibrium conditions. As computational techniques advance, the fusion of high-throughput simulations, experimental feedback loops, and AI-driven generative models will be crucial in bridging this gap, enabling more reliable predictions and rational design strategies for next-generation SEs.

#### SEs with high mechanical strength and stability

The mechanical properties of SEs are paramount to the performance and durability of SSBs. Exceptional mechanical strength and flexibility enable SEs to withstand the stresses and strains encountered during battery operation, including volumetric fluctuations that arise during charge and discharge cycles (*111*–*113*). Superior mechanical properties also contribute to the stability of SEI/CEI, mitigating the formation of cracks. SE stability can include electrochemical stability and chemical stability. Electrochemical stability refers to the ability of SEs to withstand high/low electrochemical potential drops and resist degradation under operating battery conditions (*114*–*116*). This includes resistance to decomposition and maintaining ionic conductivity over a wide voltage range, such as reduction and oxidation energetics (*E*_red_ and *E*_ox_). Chemical stability, on the other hand, involves the SE’s resistance to chemical reactions with the electrode materials and other components of the battery (*13*, *117*). Both electrochemical and chemical stability are essential for ensuring the long-term performance and safety of SSBs. Several representative AI-driven studies take mechanical strength and stability into consideration for SE screening. This section highlights the innovations of these studies.

To facilitate the rapid prediction of elastic properties in potential SEs, an ML-based framework was proposed to predict the mechanical properties, including shear modulus and elastic constant (*118*). GCNs were trained for shear and bulk modulus, and elastic constants were trained using gradient boosting regressors and kernel ridge regression, where the choice of model depends on the size of the training data and the noise it can handle. The materials stiffness is found to increase with mass density and the ratio of Li^+^ to sublattice bond ionicity and decrease with increasing per-atom volume and sublattice electronegativity. Using this framework, more than 20 mechanically anisotropic interfaces between Li metal and four SEs (Li_2_WS_4_: $P\bar{42}m$ and $I\bar{42}m$, LiBH_4_: *P*1, and LiOH: *P*4/*nmm*) were predicted that can be used to suppress dendrite growth. The screened candidate materials are generally soft and highly anisotropic, providing an opportunity to simultaneously obtain dendrite suppression and high ionic conductivity in SEs.

**Fig. 6.**
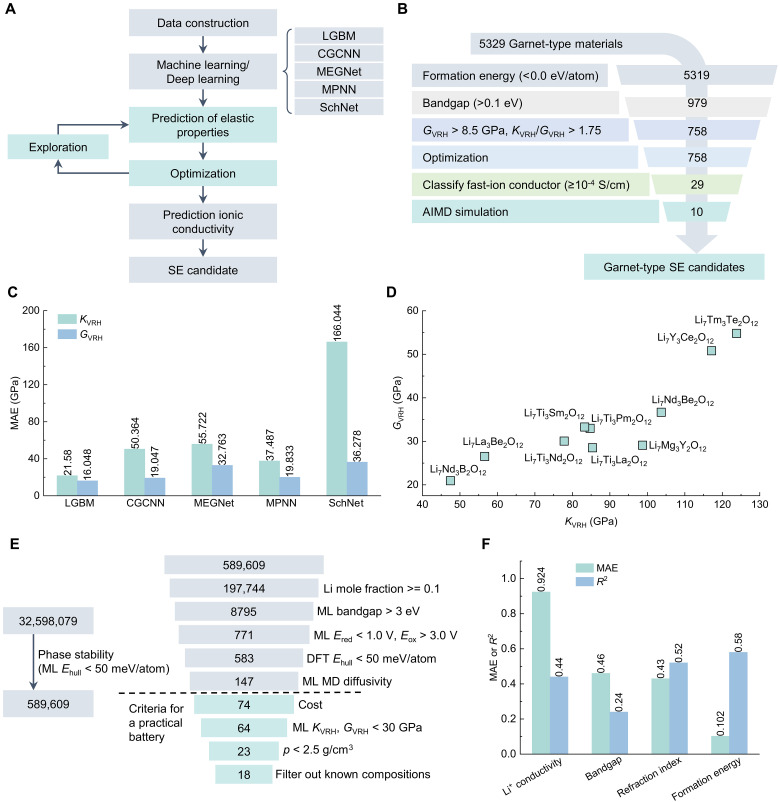
Screening SEs with high mechanical strength and stability via ML methods. (**A**) Flowchart of the ML process to predict elastic properties (bulk modulus *K*_VRH_ and shear modulus *G*_VRH_) and ionic conductivities of SE candidates (*196*). LGBM, light gradient boosting machine; CGCNN, crystal graph convolutional neural network; MEGNet, MatErials graph network; MPNN, message passing neural network; and SchNet, Schrodinger network. (**B**) Screening process to select garnet-type SE candidates (from 5329 to 10) (*121*). (**C**) Predictive MAE of each model for *K*_VRH_ and *G*_VRH_. (**D**) *K*_VRH_ and *G*_VRH_ for 10 materials validated as promising SEs. (**E**) Main screening stages of the HPC workflow for filtering candidate materials (*122*). The first stage identifies stable materials using MLFFs to perform structural relaxation and assess thermodynamic phase stabilities. The second stage is used to discover SEs with electronic and electrochemical properties. (**F**) Predictive accuracy (*R*^2^ and MAE) of four properties [Li^+^ conductivity (S/cm), bandgap (eV), refractive index, and formation enthalpy (eV/atom)] with data augmentation based on COSNet (*35*).

Similarly, an advanced ML-driven surrogate model with active learning was developed to predict the bulk modulus *K*_VRH_ and shear modulus *G*_VRH_, where VRH denotes the Voigt-Reuss-Hill approximation (Fig. 6, A and B) (*119*). The model was trained using elasticity datasets, incorporating elemental property–based and graph-based descriptors. As shown in Fig. 6C, among various ML models tested, the light gradient boosting machine (LGBM) (*120*) exhibited the highest agreement with DFT calculations, yielding mean absolute errors (MAEs) of 21.580 and 16.048 GPa for *K*_VRH_ and *G*_VRH_, respectively. Leveraging this predictive model, a systematic screening workflow was established (Fig. 6B) to discover promising SEs from garnet-type materials (*121*), encompassing 73 chemical element substitutions at the La and Zr sites in Li_7_La_3_Zr_2_O_12_. Beyond mechanical properties, additional criteria including thermodynamic stability, electronic conductivity, and ionic conductivity were integrated to refine the selection process. As a result, 10 tetragonal-phase garnet SEs, such as Li_7_Ti_3_Pm_2_O_12_ and Li_7_La_3_Be_2_O_12_ (Fig. 6D), were selected and subsequently verified to have superior mechanical strength and suitable conductivity. While this study primarily focuses on garnet-based electrolytes, it underscores the broader applicability of ML in accelerating the prediction of mechanical properties across various SE classes, offering valuable guidance for the rational design of next-generation SEs.

Further advancements in computational materials discovery have been realized through the integration of ML models with physics-based simulations, deployed on cloud-based high-performance computing (HPC) platforms (*122*). A comprehensive screening workflow has been developed to assess materials stability and SE performance by leveraging cloud-based infrastructure. The discovery process unfolds in two sequential stages (Fig. 6E): first, stable materials are identified through structural relaxation using MLFFs, followed by thermodynamic phase stability assessments via Pymatgen (*63*); second, a rigorous filtering process is applied to pinpoint materials that satisfy key electrochemical criteria, including band gaps, *E*_red_, *E*_ox_, *E*_hull_, and ionic diffusivity. Additional practical considerations such as cost, *K*_VRH_, and *G*_VRH_ are also incorporated. This end-to-end workflow seamlessly revolutionizes SE discovery and accelerates materials identification, synthesis, and experimental validation. More importantly, HPC platforms enhance computational efficiency by optimizing resource utilization and enabling faster processing of complex tasks, reducing energy footprints through techniques like efficient hyperparameter tuning, parameter sharing, and scalable parallelization, aligning computational advancements with sustainable practices.

Multimodal ML has further expanded the predictive capabilities in materials science through a composition-structure bimodal learning framework, termed the Composition-Structure Bimodal Network (COSNet) (*35*). This approach synthesizes information from both chemical composition (text-based descriptors) and crystal structure (graph-based representations) to construct a unified materials descriptor. COSNet has demonstrated superior performance compared to conventional single-modal models that rely exclusively on either composition- or structure-based features in predicting Li^+^ conductivity, electronic conductivity, refractive index, and formation energy for SEs (Fig. 6F). By integrating COSNet with AIMD simulations, a high-throughput pipeline for SEs has been established, leading to the identification of several Li^+^ conductors with ionic conductivities exceeding 0.1 mS/cm at room temperature. The materials found span diverse chemical compositions, including oxides, halides, and mixed-anion compounds. This study underscores the transformative potential of multimodal ML in addressing challenges associated with data scarcity and sparsity in materials informatics. In addition, it highlights the necessity of a multifaceted approach when screening SSEs, as multiple interdependent materials properties, such as mechanical strength, stability, and conductivity, must be simultaneously optimized to achieve superior electrochemical performance.

Combining AI-aided screening or doping processes with experimental validations reduces the high workload associated with the trial-and-error approach for designing SEs with mechanical properties and stability. For instance, the BO method has been applied to the (Li*_y_*La_(1–*y*)/3_)_1–*x*_Sr_0.5*x*_NbO_3_ (0 ≤ 0.5*x* ≤ 0.15, 0 ≤ *y* ≤ 0.3) composition for searching the best compositions with superior sintered morphologies (*123*). It was found that the BO method requires an average of 20 observation steps to find the optimal solution in the dataset, with a success rate of more than 99%. Compared with the random search method, the BO method shortens the number of steps required by two-thirds and improves the efficiency of experimental design. This provides a reference for the real implementation of AI modeling in designing high-performance SEs.

Overall, ML-driven research on the mechanical properties, electrochemical stability, and chemical stability of SEs remains underdeveloped compared to studies focused on ionic conductivity. This disparity can be attributed to several key factors. First, the inherently low ionic conductivity of SEs represents the most pressing and fundamental challenge in the field, necessitating immediate solutions. Extensive experimental and theoretical investigations have already established a substantial foundation of data, providing critical support for AI-driven predictions of ionic conductivity. Second, datasets related to mechanical properties and dynamic/static disorders remain scarce, limiting the scope for robust AI modeling. Research on polymer SEs remains relatively limited compared to inorganic SEs. These aspects have yet to garner sufficient attention and engagement from the AI research community. To address the scarcity of mechanical and stability data, one effective approach is leveraging TL (*124*), where pretrained models on related datasets are fine-tuned with limited mechanical and stability data, enabling the model to generalize well despite data scarcity. GenAI models can also be used to synthesize realistic mechanical property data, augmenting the existing dataset. In addition, active learning strategies can be implemented to iteratively identify and acquire the most informative data points, optimizing data collection efforts (*125*). These methods collectively mitigate data scarcity challenges and improve the reliability of mechanical and stability property databases.

### AI-driven optimization for SEI/CEI

The interface optimization of SSBs is crucial for enhancing battery performance, as interphase issues critically influence cell resistance and eat up available materials, thereby dictating the energy density, cycle life, and safety of the battery. Concurrently, the growth of Li dendrites can grow through the SEI layer and penetrate the SE, potentially causing a short circuit or thermal runaway (*3*). Moreover, poor chemical stability at these interfaces often results in detrimental side reactions, contributing to capacity fade and reduced life span. These interfacial challenges are intrinsically linked to materials composition, microstructure characteristics, and operating conditions (*126*). However, traditional experimental approaches struggle to precisely characterize the dynamic behaviors for these interphases, and optimizing them requires a delicate balance among electronic conductivity, ionic conductivity, mechanical robustness, and chemical compatibility, further complicating research efforts (*127*–*129*). The AI-driven design provides a transformative potential for interface optimization. Leveraging ML and big data analytics, AI can expediently screen materials combinations, predict SEI/CEI behaviors, and optimize structural configuration, thereby drastically reducing the R&D timeline (*130*, *131*). Furthermore, AI can simulate intricate interfacial reaction mechanisms, propose innovative solutions, and drive technological breakthroughs in SSBs (*132*). In this section, we delve into AI-driven strategies for SEI/CEI optimization, aiming not only to accelerate interface design but also to elucidate the underlying chemical mechanism governing SEI/CEI layers (*55*, *86*, *133*–*140*), pacing the way for SSB technologies (see table S5).

#### Advances in SEI chemistry for high-capacity anodes

The SEI layer forms between SEs and the anode, preventing direct contact to minimize side reactions and extend battery life span. Its stability is particularly crucial in high-capacity anodes like silicon and Li metal. Silicon anodes, despite their high theoretical capacity, undergo volume changes during cycling, leading to SEI rupture and reformation, which degrades cycling performance. Li metal anodes, on the other hand, face the challenge of dendrite growth, which can penetrate the SEI layer, causing short circuits and safety risks. Optimizing the SEI’s chemical composition and structure is key to regulating the ionic conductivity, electronic conductivity, and mechanical properties, further enhancing the cycle life and safety of SSBs (*47*, *141*). Recent advancements in experimental and theoretical approaches have improved SEI research. Experimentally, SEI formation can be studied under simulated battery conditions using electrochemical testing, scanning electron microscopy (SEM), transmission electron microscopy (TEM), and XPS. Theoretically, AI/DFT models are used to simulate SEI formation and interfacial reactions, enabling high-precision insights into its formation mechanisms and behavior under various conditions. These simulations not only guide experimental validation but also facilitate the rational design and optimization of SEI layers. For instance, taking bulk-scale SEI-type Li_6_PS_5_Cl as a case, the kinetic predictions using a Wagner-type diffusion model align well with recent results of electrochemical studies on cell-level multiphase SEIs (*37*, *142*, *143*).

**Fig. 7.**
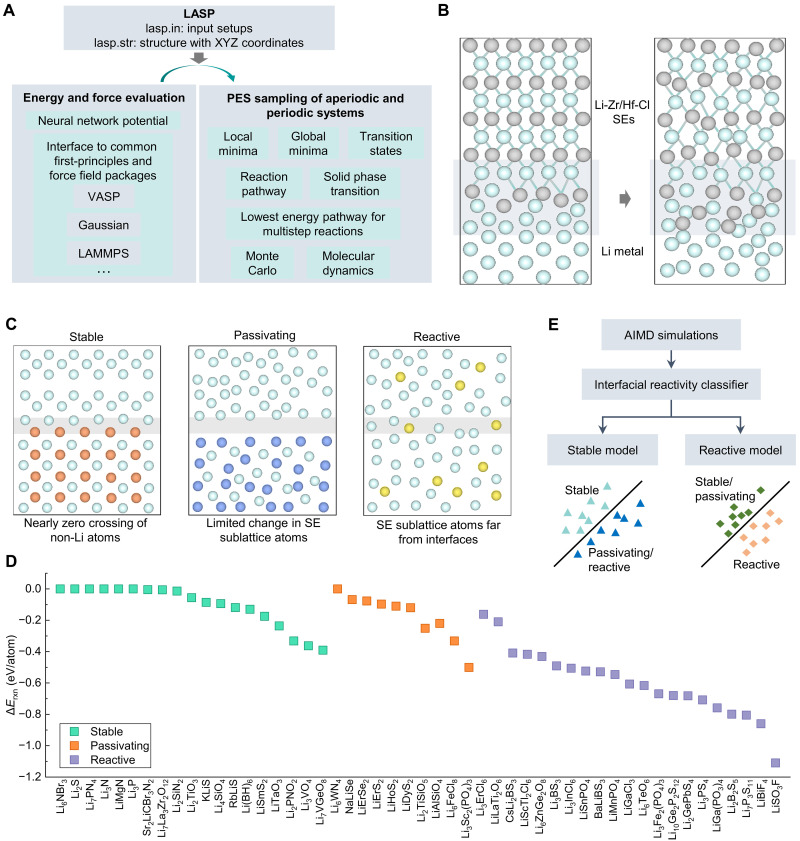
SEI investigation by combining AIMD simulations and ML methods. (**A**) Workflow of LASP (*144*): LASP takes input setups (lasp.in) and structure coordinates (lasp.str) to perform energy and force evaluations using MLFFs and interfaces with various first-principles and force field packages. (**B**) Modeling structures for Li/LiZrC or Li/LiHfC systems. MLFFs are used to conduct MD simulations based on the LASP. (**C**) Visualization and labeling of three interfaces in the ML training set (stable, passivating, and reactive) from AIMD simulations. (**D**) Labeling of SEI reactivity based on AIMD simulations. Δ*E*_rxn_ is chemical mixing energy with other battery components by DFT calculations. (**E**) Flowchart for predicting the SEI reactivity. Two classification models (stable model and reactive model) can be built to differentiate between reactive and unreactive SEI.

A highly effective approach to accelerate SEI simulation involves leveraging MLFFs to model atomic dynamics. The LASP (Large-Scale Atomistic Simulation Package) (*144*), incorporating the Stochastic Surface Walking global optimization method with a global neural network potential (SSW-NN) method, has emerged as a powerful tool for rapid structural predictions and mechanistic studies in electrodes and SEs (Fig. 7A). Recent investigations have demonstrated the capability of the SSW-NN approach in the rational design of halide SEs (*145*), unveiling a layered Li-Zr/Hf-Cl structure that enables ultrafast Li^+^ conduction and enhanced interfacial stability (Fig. 7B). Through systematic structural searcher with the Li-Zr/Hf-Cl ternary phase space, the most stable configurations were identified, followed by experimental synthesis using a mechanochemical route. The resulting Li_2_MCl_6_ (M = Zr, Hf) exhibited exceptional Li^+^ conductivity (~1 mS/cm) and outstanding interfacial stability against Li metal, sustaining symmetric cycling for up to 4000 hours. Furthermore, an SSB prototype using Li_2_MCl_6_ as SEs without any surface modifications demonstrated remarkable stability, underscoring the advantages of ML-based kinetic simulations in accelerating SEI research and guiding SE screening.

In another advancement, a computationally efficient, data-driven framework was developed to predict the reactivity of SEI formation between any candidate SEs and Li metal anodes (*133*, *134*). This model, informed by AIMD simulations, classifies SEI behavior into three categories (Fig. 7C): “stable” (minimal atomic rearrangement at the SEI), “passivating” (limited atomic diffusion within the SE sublattice), and “reactive” (structural reorganization). To enhance predictive accuracy, a dataset comprising 50 distinct SEI structures, including Li_6_NBr_3_/Li, Li_2_S/Li, and Li_7_PN_4_/Li, was curated through high-throughput simulations, capturing both kinetics and thermodynamic insights (Fig. 7D). By training two classification models on this dataset, the framework enables rapid screening of thousands for SE candidates within seconds, eliminating the need for extensive AIMD simulations (Fig. 7E). This study identified more than 300 chemically stable SEs and more than 780 passivating SEs, challenging previous thermodynamic-based assessments that often-misclassified materials as unstable. Notably, two borate-based materials, LiB_13_C_2_ and LiB_12_PC, were highlighted as promising candidates, exhibiting high ionic conductivity and robust chemical stability with Li metal, as corroborated by further AIMD evaluations. These findings suggest that the pool of viable SEs may be far larger than previously anticipated, paving the way for more efficient and accurate screening methodologies in SSB research. While the reactivity prediction model demonstrates promising accuracy using small-sample AIMD data (<10^3^), its generalizability may face limitations due to potential overfitting and insufficient chemical diversity. Small-sample training reduces computational costs and enables rapid prototyping, yet it risks missing critical reaction pathways or outliers (*146*). Mitigation strategies such as learning curve plots and uncertainty estimates can be used to test the generalization ability of few-shot models. Future work should incorporate active learning or hybrid datasets to enhance robustness while retaining efficiency (*147*). For instance, in an active learning framework, the model can iteratively identify compositions or configurations with the highest prediction uncertainty and selectively acquire high-fidelity data to refine its predictions. In a hybrid dataset approach, computational and experimental data can be combined through appropriate weighting or domain adaptation to capture both the breadth of simulations and the realism of measurements. This balance is crucial for reliable AI-driven reactivity predictions in electrolyte design.

**Fig. 8.**
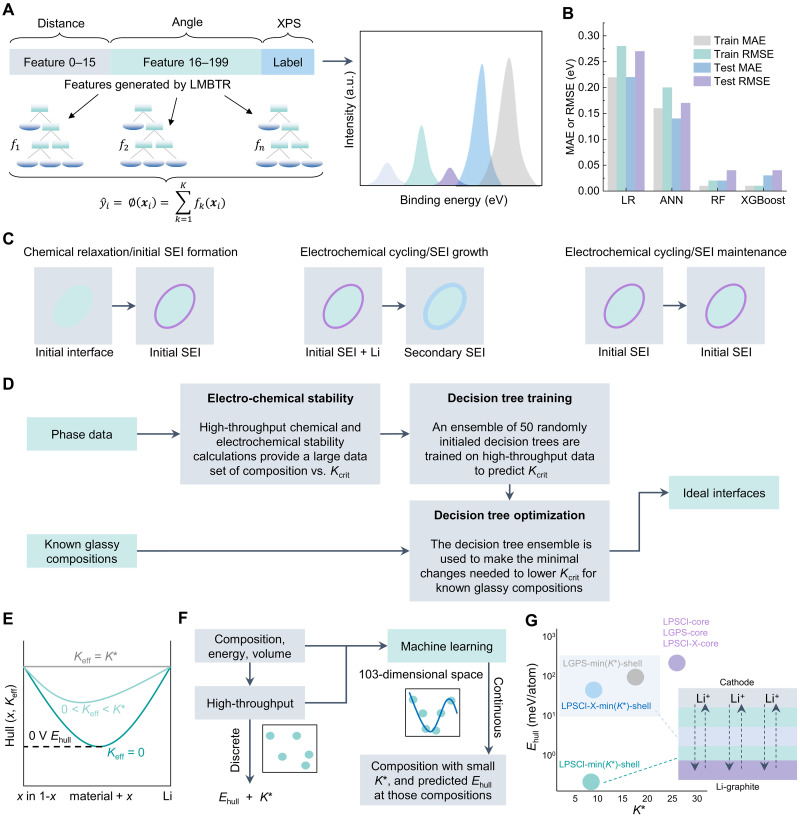
Predicting SEI properties by using ML methods. (**A**) Scheme of XPS prediction using ML models (*135*). The features of the structure can be generated using local many-body tensor representation (LMBTR) (*148*). The specified system simulated is LiFSI in DME and TTE. (**B**) RMSE and MAE values of four ML models [linear regression (LR), random forest (RF), extreme gradient boosting (XGBoost), and artificial neural network (ANN)] for predicting the C_1*s*_ XPS values. (**C**) Comparison of the different chemical and electrochemical reactions that can occur during battery assembly (left) and cycling (right). The illustrations below the table show how the electrolyte (light brown) and active material (blue) react to form a stable or unstable SEI depending on mechanical constriction. The initial SEI is in dark brown. (**D**) Overview of computational approach for predicting *K*_crit_. High-throughput computation is performed to obtain data points on how *K*_crit_ varies with composition. The RF model was then leveraged to train and predict *K*_crit_. Last, using known glassy compounds with high Li^+^ conductivity, the RF model was used to make minimal compositional changes to lower *K*_crit_. (**E**) Schematic illustration of *E*_hull_ and K^*^ in the reaction between a material and the Li metal (*137*). *K*_eff_ denotes the local effective moduli, *x* means the mixing ratio. (**F**) Schematic flowchart of AI computational procedure for predicting *E*_hull_ and K^*^. (**G**) Distribution of a few representative electrolyte compositions in the *E*_hull_-K^*^ space. LPSCl-core and LGPS-core were predicted by ML without minimizing K^*^. LPSCl-X-core includes the original compositions of Li_5.5_PS_4.5_Cl_1.1_F_0.4_, Li_5.5_PS_4.5_Cl_1.35_Br_0.15_, and Li_5.5_PS_4.5_Cl_1.35_I_0.15_. LPSCl-min(K^*^)-shell was predicted from the ML minimization of K^*^. LGPS-min(K^*^)-shell was minimized from the LGPS-core original composition to give the modified composition of Li_14.2_Ge_0.9_P_1.8_S_8_. The region of LPSCl-X-min(K^*^) includes the minimizations from the LPSCl-X-core compositions, giving Li_7.9_P_0.5_S_3.1_Cl_0.7_F_0.25_, Li_7.1_P_0.5_S_2.4_Cl_2.2_Br_0.375_, and Li_7.2_P_0.5_S_2.3_Cl_2.2_I_0.375_, respectively.

As mentioned above, XPS is a powerful surface analysis technique extensively used to characterize SEI layers. However, conventional XPS measurements are inherently limited in probing the atomic structure of deeply buried SEI layers, omitting crucial details about their composition and evolution. To overcome this challenge, a computational framework (Fig. 8A) integrating hybrid DFT, reactive MD, and ML models was developed to predict the XPS spectra of SEI (*135*). The study specifically examined Li bis(fluorosulfonyl)-imide (LiFSI) in a solvent system comprising dimethoxyethane (DME) and 1,1,2,2-tetrafluoroethyl-2,2,3,3-tetrafluoropropylether (TTE), with a focus on the C_1*s*_ binding energy due to the diversity and significance of carbon-containing species in SEI chemistry. A local many-body tensor representation (LMBTR) was used to describe SEI structures (*148*), enabling the training of four predictive models for core-level shifts (Fig. 8B). Among them, extreme gradient boosting (XGBoost) achieved the highest accuracy, with an MAE of 0.03 eV and a root-mean-squared error (RMSE) = 0.04 eV on test data. By enabling precise XPS predictions, this methodology provides deeper insights into SEI composition and structure, which are essential for optimizing battery materials and interfacial engineering. Moreover, the data based on time-of-flight secondary ion mass spectrometry (ToF-SIMS) can be used to identify Li compounds (Li_2_CO_3_, Li_2_O, Li_3_N, LiH, and LiOH) on Li metal anode with ML classification models, providing a potentially way to investigate the multicomponent of SEI (*149*, *150*).

The mechanical evolution of SEI under cycling conditions is another critical aspect influencing the stability and longevity of SSBs. The schematic in Fig. 8C illustrates how the SE (light gray) and active material (green) react to form a stable or unstable interface, contingent on the mechanical constriction at the interface. When the effective local modulus (*K*_eff_) remains below a critical threshold (*K*_eff_ < *K*_crit_, defined by the interfacial materials), this SEI continues to react and grow into an unknown phase (blue). Conversely, when *K*_eff_ > *K*_crit_, the interface remains structurally intact. Because mechanical constraints are typically absent during initial SEI formation, the interphase develops in an unconstrained environment, whereas subsequent cycling conditions may or may not impose mechanical confinement, influencing SEI evolution and stability over extended operation. To enable systematic investigation and design of SEI structures with tunable electrochemical stability, a high-throughput DFT-based workflow integrated with a random forest model was developed (*136*). This study explored more than 80,000 SEI compositions via DFT calculations, combining more than 20,000 electronic insulators from the MP database with four widely studied ceramic-sulfide SEs: Li_10_GeP_2_S_12_ (LGPS), Li_10_SiP_2_S_12_ (LSPS), Li_7_P_3_S_11_ (LPS), and Na_7_P_3_S_11_ (NPS). A random forest ensemble was trained to predict the ground-state phase *K*_crit_ for any given composition (Fig. 8D). The model was then applied to refine the composition of a parent glassy-phase sulfide SE with high Li^+^ conductivity, optimizing the SEI to minimize *K*_crit_. Experimental validation confirmed that the engineered SEI undergoes controlled decomposition and electrochemical passivation consistent with the ML predictions, reinforcing the effectiveness of data-driven interfacial engineering. This approach establishes a systematic framework for studying SEI structures in all-solid-state electrochemical systems, highlighting the critical role of interfacial modulus tuning in stabilizing dynamic interfaces.

To achieve stable cycling in SSBs, the penetration of Li dendrites and the subsequent microcrack propagation at high current densities represent critical challenges. Recent advancements have leveraged SEI decomposition reactions as a mechanism to suppress Li dendrite penetration through a “dynamic stability” effect. In this context, an innovative approach has been introduced to optimize SEs for SEI formation by strategically positioning them within a two-parameter design space defined by *E*_hull_ and *K*_eff_ (Fig. 8E) (*137*). This framework establishes that only SEs occupying specific regions of this design space can effectively mitigate dendrite-induced degradation. Using DFT simulations from the MP database, the study extracted materials information on composition, energy, and volume under unconstrained conditions (*K*_eff_ = 0 GPa). Subsequently, the *E*_hull_ at 0 eV was computed as a function of *K*_eff_ ≥ 0 GPa, while the critical modulus (K^*^) values were determined for 124,497 materials at SEI layers. A decision tree model was then used to correlate the macroscopic properties of composition and energy with the target values of *E*_hull_ and K^*^, enabling the extrapolation of this relationship across a continuous compositional space. This predictive capability allows for systematic compositional optimization, driving the minimization of K^*^ for any given compositions. The study further mapped the distribution of several key electrolytes within the *E*_hull_–K^*^ phase space, as predicted by the ML model (Fig. 8F). Notably, pristine Li_5.5_PS_4.5_Cl_1.5_ (LPSCl), Li_10_GeP_2_S_12_ (LGPS), and Li_5.5_PS_4.5_Cl_1.5–*y*_X*_y_* (LPSCl-X, where X = F, Br, or I, with *y* = 0.4 for F and 0.15 for Br and I, respectively) all exhibit high K^*^ (>20 GPa) and *E*_hull_ values (>150 meV) without K^*^ minimization (the “core” compositions in Fig. 8G). This positioning suggests that such homogeneous electrolyte particles may not inherently provide optimal dynamic stability. In contrast, ML-driven compositional modifications of LPSCl, LGPS, and LPSCl-X [min(K^*^)-shell compositions in Fig. 8G] shifted in phase space toward lower K^*^ based on ML minimization of K^*^. The optimized compositions were observed to be Li rich and S deficient compared to their pristine counterparts, reinforcing the efficacy of targeted composition tuning. This work demonstrates a robust materials design strategy wherein dynamic stability against Li dendrite formation and penetration can be systematically engineered by navigating the two-dimensional design space defined by *E*_hull_ and K^*^. By directing material compositions toward moderate *E*_hull_ values and minimized K^*^, this approach paves the way for the rational design of dendrite-resistant SEs.

In addition to the stability matching SEs, there are several studies demonstrating that the electronic conductivity of the SEI determines the growth kinetics with faster growth led by more pronounced electronic transport (*151*). Furthermore, electron leakage through the SEI causes the reduction of the SEs and the consumption of Li^+^, decreasing the SSB capacity and performance. Given the multicomponents and mosaic structures of SEI, the extended defects such as GBs and interfaces in SEI are likely to serve as the electron conduction pathways. Feng *et al.* (*152*) used DFT calculations to investigate the electronic properties of GBs of the main SEI components on the Li metal, suggesting that dense SEI structures such as sharp interfaces and well-ordered GBs are preferred to design a fully electronically passivating SEI. However, the SEI evolution process involving the accumulation of reduction products at the anode is difficult to simulate accurately by computations, let alone construct high-quality data for AI research (*153*).

Undoubtedly, AI has shown great advantages in the optimization and design of the interface by establishing structure-property relationships and enabling efficient MD simulations through MLFFs. By leveraging vast datasets, AI is able to identify key descriptors that govern SEI properties, such as ionic conductivity, electronic conductivity, mechanical stability, and electrochemical compatibility. MLFFs further enhance the accuracy and efficiency of MD simulations, allowing researchers to explore SEI formation and evolution at atomic scales over extended timescales. However, for the SEI engineering, the current structure-activity relationship database has not yet been formed; that is, the data on the stability, electronic and ionic conductivities, and mechanical properties of SE and anodes are scarce, which makes it a challenge for AI to directly study SEIs. Also, interphases are not homogeneous, sometimes even layered, affecting the AI/DFT modeling and experiments (*149*). In addition, research on SEI coating materials is also scarce, and whether there is a suitable coating material that can solve the interface impedance of SEI also needs further research. Using efficient MLFFs to construct a large benchmark dataset for SEI properties may be the first step; then, AI holds immense potential to further improve SEI design by integrating multiscale modeling, enabling real-time feedback loops between experiments and simulations, and accelerating the discovery of electrolyte additives or interface-modifying agents.

#### Advances in CEI chemistry for high-voltage cathodes

A well-formed CEI can reduce interfacial resistance, improve ion transport, and prevent undesirable side reactions between the cathode and the SEs. This leads to higher energy density, better cycle stability, and reduced capacity fade. However, challenges remain in achieving an ideal CEI. These include controlling the uniformity and thickness of the interphase, ensuring compatibility between materials, and mitigating degradation mechanisms such as cracking or delamination during cycling (*15*). In addition, understanding the complex chemical and electrochemical processes at the interface requires advanced characterization techniques. Addressing these challenges is key to unlocking the full potential of SSBs for next-generation energy storage.

An effective strategy to mitigate CEI degradation in SSBs involves the rational design and optimization of coating materials. These materials serve as a protective layer between the cathode and the SE, thereby suppressing detrimental side reactions and enhancing interfacial stability (*154*, *155*). The selection of appropriate coating materials, including oxides, sulfides, and polymer, has been demonstrated to extend the SSB cycle life and safety. Beyond materials selection, critical design parameters such as coating thickness, uniformity, and adhesion must be meticulously optimized to maximize functional efficacy in practical applications (*156*, *157*). For coating materials to be viable in SSB applications, they must exhibit several key attributes: high ionic conductivity, to facilitate efficient Li^+^ transport across the coating without introducing resistance; chemical stability, to prevent undesirable side reactions with both the cathode and SEs; balanced ionic and electronic conductivities, to improve the charge transfer kinetics without including the decomposition of SEs (*158*, *159*); mechanical robustness, to withstand stress and deformation during repeated charge-discharge cycles; uniformity and strong adhesion, to ensure continuous and defect-free coverage over the electrode or electrolyte interface; and processability, to enable scalable manufacturing and seamless integration into practical battery architectures (*52*, *86*).

**Fig. 9.**
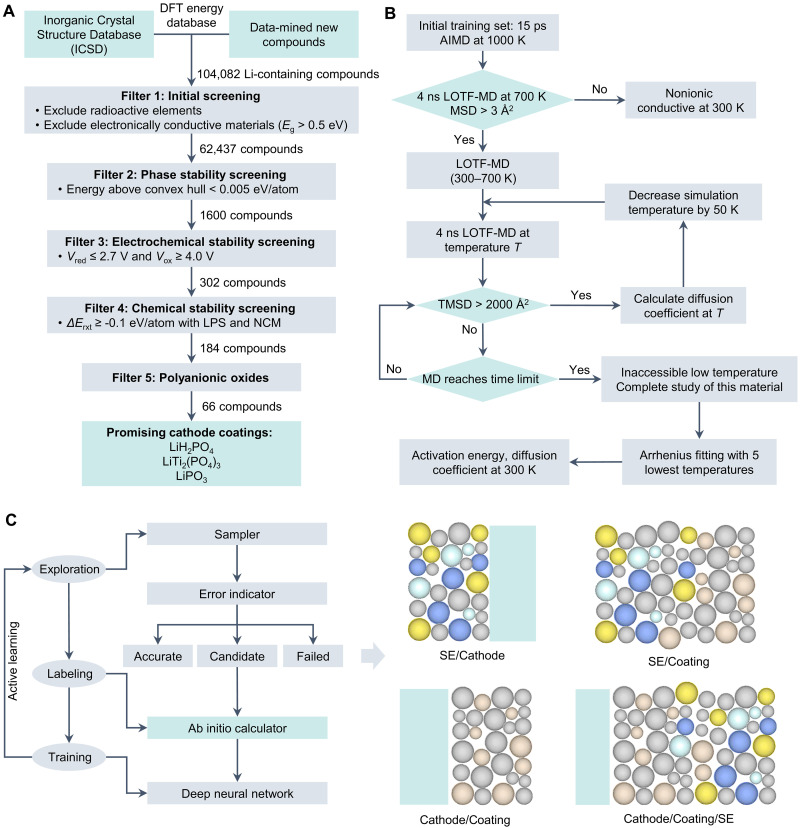
Approaches for screening coating materials for cathodes. (**A**) Flowchart describing the computational screening of cathode coating materials, following the initial screening, phase stability, electrochemical stability, and chemical stability were used as sequential filters for the high-throughput screening (*160*). *V*_red_ and *V*_ox_ are the reduction and oxidation limits of the electrochemical stability window in V versus Li metal, respectively. Δ*E*_rxt_ is the reaction energy of the material with the cathode or electrolyte in eV/atom; LPS denotes the SE Li_3_PS_4_; and NCM denotes the fully lithiated cathode material LiNi_1/3_Co_1/3_MN_1/3_O_2_. (**B**) Flowchart of the learning on-the-fly MD (LOFT-MD) simulations for screening coating materials (*138*). All potentials were pretrained by DFT data generation in AIMD simulations before being used in LOFT-MD simulations. (**C**) Investigation of effects of coating layers on the CEI by ML-assisted MD simulations (*139*). The left part provides the workflow of deep potential models (*161*), which is an active learning loop to establish a force field for predicting the energies and force of a system. The right part gives the modeling for exploring the effects of coating materials on the CEI.

Considering all these screening criteria, a computational framework was used to systematically evaluate and screen Li^+^-containing materials as potential cathode coatings (Fig. 9A) (*160*). The database used in this study comprised structures from the ICSD along with additional structures generated via chemical element substitution through data mining, culminating in a comprehensive dataset of 104,082 Li^+^-containing compounds. A multistep high-throughput screening strategy was implemented, sequentially filtering candidates based on electronic conductivity, phase stability, electrochemical and chemical stability, and ionic conductivity. Through this hierarchical selection process, polyanionic oxide coatings emerged as the most promising class of materials with LiH_2_PO_4_, LiTi_2_(PO_4_)_3_, and LiPO_3_ standing out as particularly attractive candidates. In addition, certain Li borates exhibited exceptional (electro)chemical stability across diverse interfaces, further underscoring their potential as cathode coatings in SSBs. These findings demonstrate the viability of using high-throughput computational methods for the rational discovery and optimization of coating materials.

However, identifying materials with sufficiently high Li^+^ conductivity from such an expansive materials space remains a formidable challenge. Traditional methods, such as AIMD simulations, can become prohibitively expensive when applied to materials with modest Li^+^ conductivity, which may still serve as effective interfacial coatings. To overcome this computational bottleneck, Wang *et al.* (*138*) introduced an MLFF-accelerated approach to expedite AIMD simulations. To ensure fidelity to DFT calculations, an on-the-fly learning strategy (LOFT-MD) was proposed (Fig. 9B), allowing the MLFF to dynamically refine its prediction during MD simulations to screen cathode coating materials. Using this accelerated approach, Li_3_Sc_2_(PO_4_)_3_ and Li_3_B_7_O_12_ were identified as highly promising battery coating materials. In addition, several other candidates (e.g., Li_3_AlF_6_, Li_2_B_3_O_4_F_3_, and LiLuF_4_) were recognized for their potential as dopants to enhance ionic conduction. This work highlights the transformative role of MLFF-accelerated simulations in materials discovery, offering an efficient and scalable pathway for optimizing interfacial coatings.

The interfacial impedance escalation in SSBs predominately originated from spontaneous (electro)chemical reactions and restricted Li^+^ diffusion kinetics. This fundamental trade-off necessitates meticulous optimization in coating materials design. Notably, ML-assisted MD simulations have recently elucidated the structural reconstruction dynamics and Li^+^ transport characteristics at LiCoO_2_/Li_6_PS_5_Cl interfaces mediated by amorphous LiF (a-LiF) coatings (*139*). Before extended MD simulations, researchers rigorously validated MLFFs through comparative analysis between DFT + U and deep potential (DP) models (*161*). The optimized DP potential demonstrated exceptional predictive accuracy (energy RMSE: of 2.5 meV/atom; force RMSE: 109.7 meV/Å) on the benchmark datasets (Fig. 9C). Subsequent large-scale DP-MD simulations of LiCoO_2_/a-LiF, LiCoO_2_/Li_6_PS_5_Cl, Li_6_PS_5_Cl/a-LiF, and LiCoO_2_/a-LiF/Li_6_PS_5_Cl revealed critical coating functionality: While a-LiF effectively preserves P-S tetrahedron configurations in Li_6_PS_5_Cl, it exhibits limited capacity to suppress S_2_ dimer formation. Crucially, a thickness-dependent transition in Li^+^ mobility was identified, with ordered structural domains emerging beyond the optimal 1-nm coating threshold, thereby impeding ionic conduction. These atomistic insights establish fundamental design principles for CEI engineering.

**Fig. 10.**
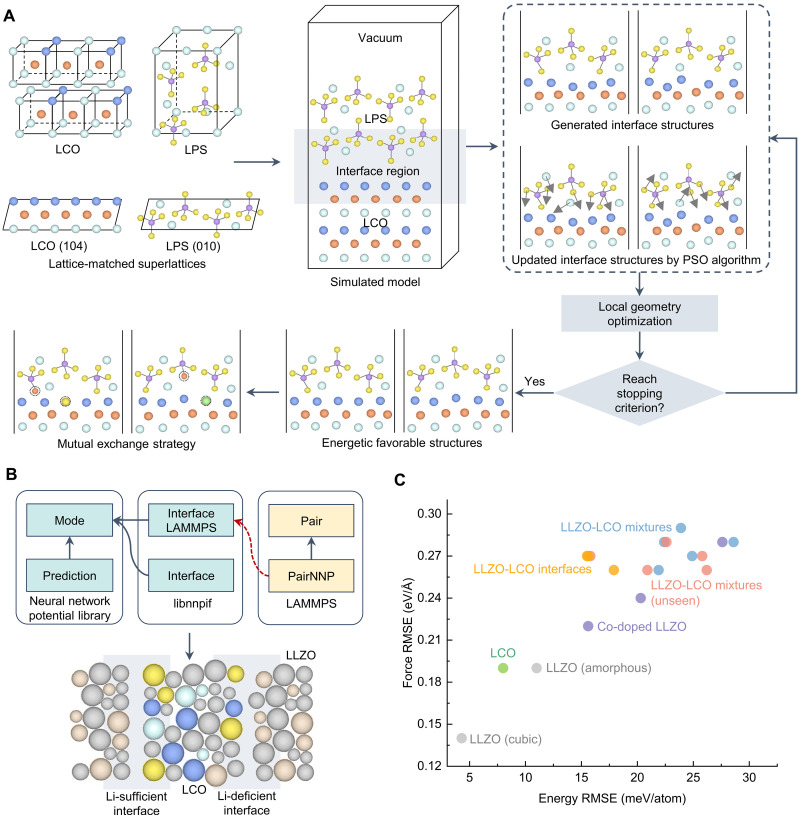
AI-assisted modeling for CEI investigation. (**A**) Flowchart for the prediction scheme of heterogeneous interface structures using the CALYPSO methodology (*162*). LiCoO_2_ (LCO) and β-Li_3_PS_4_ (LPS) were selected as cathode and SE (*140*). Then, a lattice-matched superlattice for the LCO(104)/LPS(010) interface was determined, and the local atomistic structure in the interface region was explored by the CALYPSO method. (**B**) Schematic overview of class inheritance for a neural network potential package (n2p2) (*55*). Gray lines point from inherited to base classes, and the red dotted line indicates that the PairNNP instantiates the LAMMPS (Large-scale Atomic/Molecular Massively Parallel Simulator) interface. On the basis of the n2p2, the interface of LCO/ Li_7_La_3_Zr_2_O_12_ (LLZO) was then explored (*163*). (**C**) RMSEs of energies and forces of snapshots from the ML-assisted simulations. Gray points mean the LLZO structures (cubic and amorphous), green point represents the layered LCO structure, purple points are Co-doped amorphous LLZO structures, blue and red points indicate the known LLZO-LCO mixtures and unseen LLZO-LCO mixture, respectively, and orange points are the LLZO(001)-LCO(104, 100, 110) interfaces.

Parallel advancements in AI-driven interface characterization have enabled mechanistic investigations of Li^+^ transport phenomena. A representative study using swarm intelligence–enhanced particle swarm optimization (PSO) with DFT validation systematically analyzed LiCoO_2_ (LCO)/β-Li_3_PS_4_ (LPS) interfacial dynamics (Fig. 10A) (*140*). In the study, a heterogeneous LCO/LPS CEI was first determined, and the interface structures were sampled and updated by the PSO method using the CALYPSO package (*162*). Then, using DFT calculations, the favorable CEI structures were obtained for analysis. Crystal structure analysis of heterogeneous interfaces revealed cation-anion intermixing (Co-P-O-S coordination) within reaction layers. Computational mapping of Li chemical potential μLi(r) and the migration energy landscape demonstrated that high μLi(r) interfacial sites induce dynamic Li^+^ depletion during charge initiation, triggering SE oxidation decomposition. These findings provide theoretical validation for interfacial resistance mechanisms and elucidate the functional basis of oxide buffer layers (e.g., LiNbO_3_) in resistance mitigation.

Beyond the high-precision modeling and analysis of static CEI structures, the dynamic evolution of CEI layers can be explored through MLFFs. Researchers leveraged the neural network potential package (n2p2) to construct MLFFs for various systems, including Li_7_La_3_Zr_2_O_12_ (LLZO), Co-doped LLZO, LiCoO_2_ (LCO), and LLZO/LCO interface (Fig. 10B) (*55*). n2p2 is a widely adopted package for computing high-dimensional NN potentials in physics and chemistry (*163*). It enables the use of pretrained NN potentials to predict energies and forces, either as stand-alone tools or integrated with MD software such as LAMMPS (*164*) By efficiently simulating and predicting atomic-level behaviors, n2p2 proves particularly suitable for studying the ion transport mechanisms at the CEI layers. The RMSE of energies and forces, as depicted in Fig. 10C, confirm the high accuracy of the training MLFFs. Leveraging this model, the onset of interfacial degradation at the garnet LLZO/LCO interface was systematically examined. By exploring various interface geometries and compositions, it was found that the Li-deficient interfaces exhibit severe interfacial disorder, accompanied by cation mixing and interdiffusion of Co from LCO to LLZO. In contrast, the Li-sufficient interface demonstrates a lower degree of disorder, albeit with localized elemental segregation. Moreover, owing to Co interdiffusion, Co-rich regions preferentially form at LLZO GBs driven by cation segregation and trapping effects. Notably, this Co-aggregation phenomenon appears independent of the GB tilt axis, GB disorder, and the Co concentration, suggesting that Co accumulation at GBs is a universal phenomenon in polycrystalline LLZO, This, in turn, profoundly influences the overall Li^+^ transport properties and mechanical stability of the material. These findings provide crucial insights into the mechanistic origins of experimentally observed physicochemical properties and offer a rational design strategy to mitigate interfacial degradation, thereby improving the cycling stability and performance of SSBs.

Similarly, to elucidate the key factors limiting Li^+^ transport across the LiNi_0.90_Co_0.05_Mn_0.05_O_2_ (Ni90)/SE interface, the microscopic processes governing Li^+^ conduction at the interface and charge transfer on the Ni90 cathode surface were carefully studied. Chen and Ong (*165*) used the universal ML interatomic potential (M3GNet) to model the interfaces of Ni90 with Li_2_S, Li_3_PO_4_, Li_2_ZrO_3_ (LZO), and Li_3_InCl_6_ (LIC). Analysis of Li site energy distributions at these CEI layers revealed that Ni90-LZO and Ni90-LIC present relatively small increases of Li site energies, whereas Ni90-Li_2_S and Ni90-Li_3_PO_4_ interfaces exhibit prominent high-energy barriers for Li^+^ transport (*86*). The larger energy barrier at the Ni90-Li_3_PO_4_ interface is attributed to the lattice mismatch (~5%) between the two bulk phases. Conversely, the higher barrier at the Ni90-Li_2_S interface is primarily due to the distinct anionic coordination environments surrounding Li^+^ on either side of the interface. Specifically, Li^+^ in Li_2_S is coordinated and predominated by S^2−^, whereas on the Ni90 side, Li coordination is primarily dominated by O^2−^. This mismatch in local anionic environments leads to higher site energies and a less uniform Li distribution compared to fully coordinated Li sites within the respective bulk phase. In contrast, the Ni90-LZO interface exhibits a lower barrier, likely due to the similar Li coordination environments composed of O^2−^ and transition metals on both sides. In addition, the low energy barrier at the Ni90-LIC interface is attributed to the structural flexibility of LIC, where Cl^−^ anions adopt a cubic packing arrangement. This contrasts with the densely packed anions in Li_2_S (face-centered cubic), Li_3_PO_4_, and LZO (hexagonal close-packed), which restrict atomic relaxation and interfacial accommodation. Consequently, the more open structure of LIC allows for better structural adaptation to the Ni90 surface, facilitating enhanced Li^+^ transport kinetics across the Ni90-LIC interface. By integrating ML-assisted MD simulations, this study provides fundamental insights into the interfacial Li^+^ transport mechanisms and offers strategic guidance for engineering CEI interfaces to optimize ionic conductivity and electrochemical stability.

AI has emerged as a powerful tool in optimizing and designing CEI layers by uncovering complex structure-property relationships and enhancing atomic-scale simulations through MLFFs. By harnessing large-scale experimental and computational datasets, AI can identify critical factors influencing CEI stability, ionic transport, and electrochemical robustness. MLFFs enable highly efficient MD simulations, allowing for in-depth exploration of CEI formation, degradation, and interfacial evolution over extended timescales. Compared with the research on SEI, the situation of CEI may be more complicated because a variety of cathode materials are introduced instead of relatively simple Li metal or silicon. Simplifying the problem and narrowing the materials space can make the screening of SE and cathode materials independent, and then comprehensively study the interface properties. Identifying universal coating materials using AI offers a promising alternative to separately optimizing CEI interfaces. By leveraging high-throughput screening and generative models, AI can predict materials with dual compatibility, ensuring both interfacial stability with SEs and electrochemical/structural compatibility with cathodes. Such AI-driven materials discoveries could streamline battery manufacturing, reduce interfacial degradation, and enhance overall SSB longevity.

### Limitations and potential solutions

In the preceding sections, we critically reviewed recent advancements in AI methods applied to the design of SEs and the optimization of SEI/CEI layers. These studies primarily include the development of structure-property relationship-based screening pipelines, MLFFs for MD simulations, and assessing key properties such as ionic conductivity, mechanical strength, and stability of SEs. In addition, ML approaches have been used to evaluate the ion transport mechanisms and electrochemical stability of SEI/CEI layers. Collectively, these studies underscore the pivotal role of AI in SSB research. Despite these promising developments, ML applications in SSBs remain in their early stages. Will AI become a key breakthrough in accelerating the innovation of SSBs? In this section, we conduct an in-depth analysis of the major challenges and potential solutions associated with AI-driven SE and SEI/CEI design.

#### Limitations

##### Limitation 1: Modeling complex multi-scale structure-property relationships

The performance of SE and SEI/CEI is governed by multiscale factors such as electronic structure, ion transport mechanism, and phase stability. Because materials properties emerge from intricate interactions spanning atomic (chemical bonds and electronic structure), nanoscopic (defects and phase interfaces), mesoscopic scale (multiphase complexity), and macroscopic scale (mechanical properties and stability), these factors collectively dictate the functionality of SSBs. However, existing modeling approaches struggle to comprehensively capture the structure-property relationship across scales. While AI models can predict local ionic conductivity or interfacial stability, translating these predictions into real-world battery performance requires experimental validation at the device level.

One of the inherent difficulties lies in the complex structure-performance relationship, where ion transport properties depend not only on the LCE (such as coordination number and electronic structure) but also on disorder factors and strain affects (*166*). For example, fast-ion conductors like Li_10_GeP_2_S_12_ exhibit low-energy Li^+^ migration channels embedded within their crystalline frameworks, a feature that cannot be fully captured by conventional structure descriptors (*167*). The anion disorder in argyrodites represents an average disorder but still influences the ion transport. This, in turn, affects both the static and dynamic Li^+^ disorder (*168*). Despite advancements in AI-driven materials discovery, current models still struggle to fully account for such multiscale transport mechanisms. Most AI approaches rely on static structural descriptors, which often fail to capture the effects of average static disorder, dynamic disorder, anharmonic vibrations, and local strain fields, all of which play a critical role in real-world Li^+^ transport. Furthermore, the interplay between short-range interactions (e.g., LCE effects) and long-range collective dynamics remains challenging to encode in AI models trained predominantly on equilibrium structures. In addition, most AI models operate at isolated scales, leveraging either DFT calculations or AIMD simulations, without a unified predictive approach that bridges atomic, nanoscale, and macroscopic phenomena. Moreover, AI-driven insights often rely on high-quality training data, necessitating precise synthesis and characterization techniques, such as in situ spectroscopy or atomic-resolution imaging, to refine model accuracy. In a word, how to effectively incorporate these multiscale dependencies into AI models remains unresolved. Bridging this gap will require integrating data-driven representations with physics-informed models that explicitly incorporate disorder and transport dynamics, enabling a more accurate and generalizable prediction framework.

##### Limitation 2: Accurately simulating SEI/CEI thermodynamics and kinetics

The SEI/CEI layer plays a crucial role in regulating ion/electron transport and stabilizing solid-solid interfaces. However, interphase dynamics are highly complex, involving (i) electron and ion diffusions, and penetration mechanisms; (ii) thermodynamic and kinetic stability of interfacial reactions; (iii) electrochemical degradation, element migration, and interfacial reconstruction; and (iv) the influence of mechanical stress on interfacial structures. Given the multiphase nature of the products, coupled with its chemical complexity and long-term evolution, conventional computational methods such as DFT and MD struggle to accurately capture interfacial dynamics and kinetics. While ML accelerates simulations, challenges remain, including data scarcity and limited model generalization, making precise prediction of interface behavior a major bottleneck.

These challenges stem from both the intricate chemistry of interfaces and the scale limitations of computational modeling. SEI/CEI layers often comprise diverse materials (e.g., oxides, electrolytes, and metal compounds), with interfacial interactions that are difficult to model comprehensively. For instance, at the LiPON-Li interface, Li^+^ migration is influenced not only by interfacial chemistry but also by local electric fields and mechanical stress (*169*). Even when composed of the same material, interfaces formed under different processing conditions can exhibit vastly different stability and ion transport characteristics. Computationally, DFT excels at capturing interfacial electronic structures and surface energies but is limited to small systems (<1000 atoms) and short timescales (<1 ns). Classical MD simulations can describe ion migration but often lack the accuracy needed for interfacial reactions and structural reconstructions due to the limitations of force fields.

Furthermore, interface stability is not solely determined by instantaneous chemical potential and ion/electron transport behavior but is also governed by long-term structural evolution, such as element diffusion, phase transitions, and interface coarsening. For example, at the LiCoO_2_ (LCO)/LLZO interface, Co diffusion can lead to enrichment within LLZO, compromising interfacial stability and degrading ion transport properties (*55*). Such degradation typically unfolds over hundreds to thousands of charge-discharge cycles, whereas existing computational methods can only simulate nanosecond-scale behavior. This gap in timescale coverage presents a fundamental challenge in predicting long-term interfacial evolution. Moreover, when Li_1.3_Al_0.3_Ti_1.7_(PO_4_)_3_ (LATP) comes into contact with Li metal, Ti^4+^ in LATP is reduced to Ti^3+^, leading to the formation of a mixed ionic-electronic conducting interphase. This results in the induction of side reactions in SEI/CEI layers (*170*). Therefore, the interphase degradation products and their ion/electron transport properties need to be investigated to predict growth rates.

##### Limitation 3: Speeding up the efficient and green development of SSBs in the era of AI

Establishing an intelligent, end-to-end AI-driven ecosystem for SSBs would accelerate the discovery of SEs and the optimization of SEI/CEI, driving both the commercialization of SSBs and advancements in sustainable energy technologies. Unlike catalysis and drug discovery, which have already developed closed-loop platforms integrating AI, computational simulations, and high-throughput experimentation, the field of SSBs still lacks such intelligent research infrastructure. The absence of a unified, data-driven platform hampers progress, limiting the efficiency of materials discovery and interface optimization.

One of the fundamental challenges is the scarcity of high-quality, standardized materials datasets, making it difficult to train robust and generalizable AI models. In catalysis, large-scale curated databases such as the Open Catalyst Project (*171*) have enabled AI-driven catalyst discovery. In contrast, existing data for SEs and SEI/CEI remain fragmented, inconsistent in quality, and highly sensitive to external conditions such as humidity, temperature, and synthesis methods. For instance, the reported ionic conductivity of argyrodites varies by two to three times across different experiments (*172*), making it challenging for AI models to establish reliable structure-conductivity correlations. In this case, it may be more accurate to use activation energy barriers to build AI models. Computational datasets, while valuable, primarily focus on static properties like phase stability and mechanical strength, lacking critical insights into long-term interfacial evolution, such as SEI degradation pathways. This challenge is further compounded by the inherent heterogeneity of solid-solid interfaces, where local defects, microcracks, and voids introduce complexities that are far more difficult to model than catalytic surfaces, making AI-driven stability predictions more demanding.

Furthermore, experimental automation and high-throughput methodologies remain underdeveloped in the field of SSBs. Unlike catalyst synthesis, which benefits from rapid combinatorial approaches, SE materials typically require high-temperature sintering and solid-state reactions, making automated fabrication far more challenging. SEI/CEI interface studies still rely on labor-intensive techniques such as TEM and XPS, which are not easily scalable for large dataset generation. In addition, battery performance evaluations are inherently time-consuming, as the cycle life of SSBs often requires hundreds of hours to months for reliable assessment, severely slowing down the iterative feedback necessary for AI model refinement. Overcoming these limitations will require a paradigm shift toward integrated AI-driven design, real-time computational-experimental workflows, and standardized data-sharing frameworks to bridge the gap between AI predictions and experimental validation.

#### Potential directions

##### Direction 1: Coupling multiscale modeling via MLFFs and bidirectional optimization

The development of MLFFs for molecules and crystalline materials to accurately simulate ion transport mechanisms and kinetic properties at atomic and nanoscale levels should be a potential solution. MLFFs can replace computationally expensive DFT calculations in AIMD simulations, enabling high-throughput ionic conductivity and activation barrier calculations and large-scale interface kinetic modeling. By leveraging MLFFs, long-time (>10 ns) kinetic simulations can be performed with reduced computational costs, facilitating the construction of extensive ionic conductivity and interface property databases. Moreover, MLFF-generated data can serve as a valuable training dataset for developing structure-property models, addressing the issue of data scarcity in SSB research.

Beyond accelerating AIMD simulations, MLFFs enable the expansion of periodic simulation cells to include systems with more than 10,000 atoms, a strategy that has been successfully validated in 100,000 disordered silicon atoms (*173*). Furthermore, MLFFs enable accurate calculation of local stress distributions at the atomic level, which are essential inputs for initiating and guiding damage evolution in continuum-level simulations. These stress fields, once spatially average or interpolated, can serve as driving forces in phase-field formulations to simulate crack initiation and propagation across materials interfaces (*174*), For instance, the atomic arrangements and diffusion coefficients from MD simulations can be used as initial conditions to study microscale evolution processes. The insights gained at the microscale, such as GB interactions and defect evolution, ultimately influence macroscopic properties, including mechanical stability and thermal management. Such a hybrid approach enhances the fidelity of crack path predictions, particularly in the battery materials systems. To fully bridge these scales, outputs from mesoscopic modeling, such as stress field distributions and defect patterns, must be integrated into finite element models (*37*, *175*) to predict the overall battery performance under operational conditions. This holistic, cross-scale coupling of atomic, nanoscale, mesoscopic, and macroscopic simulations is crucial for establishing a unified, predictive framework, facilitating more physics-informed multiscale simulations.

Multiscale modeling may introduce nonuniform data, as variations in measurement conditions, sample purities, and synthesis methods can introduce inconsistencies, making it crucial to incorporate uncertainty quantification and data curation techniques. One approach is to develop a “minimal benchmark dataset” by selecting well-characterized SE samples with consistent synthesis routes, uniform measurement setups (e.g., impedance spectroscopy under controlled conditions), and clearly documented metadata. Such datasets can serve as a reference for training and validating AI models, ensuring reproducibility and facilitating meaningful cross-study comparisons.

In addition, bidirectional feedback optimization is essential for reinforcing structure-property relationships across scales. Multitask learning offers a powerful means of handling different scales within a single model (*176*, *177*), where individual tasks can be assigned to atomic-level structure optimization and macroscopic battery performance predictions, respectively. By enabling information sharing and transfer between tasks, multitask learning enhances both the efficiency and accuracy of cross-scale modeling, ultimately advancing the design and optimization of next-generation SSBs.

##### Direction 2: Developing multimodal models embedded with physical constraints

The dynamic and kinetic evolution of SEI/CEI exhibits intricate electrochemical-mechanical coupling phenomena that conventional continuum-based approaches struggle to accurately characterize. To address this challenge, physics-informed neural networks directly incorporate embed physical constraints or prior knowledge (*178*, *179*), notably the Butler-Volmer electrochemical kinetics and mass conservation laws (*180*), into the neural architecture’s loss function, enabling physics-constrained prediction of interfacial evolution. The critical innovation lies in the model’s capacity to autonomously learn physically admissible evolution trajectories from limited experimental datasets through embedded domain knowledge.

Atomic-scale mechanism understanding of interfacial dynamics, particularly Li^+^ migration pathways and vacancy diffusion mechanisms, remains pivotal for performance optimization. Beyond the above-mentioned MLFF-based MD simulations, GNNs offer a transformative framework by representing interfacial atomic configurations as dynamic graphs. Node features encapsulate quantum chemical descriptors (e.g., partial charges and coordination numbers), while edge attributes dynamically encode bond length/angle variations, thereby capturing spatiotemporal correlations in interfacial reaction dynamics through continuous graph structure updates. These approaches would be helpful to explore the interphase degradation products and their ionic-electronic transport properties.

The emergence of GenAI has revolutionized inverse design paradigms for interface engineering. By establishing a structural phase space encompassing critical interfaces (SE/Li, SE/LCO, and SE/coatings), these generative architectures enable the property-driven synthesis of interface configurations. Leveraging MLFF-constructed material databases, such models can systematically explore the design space for interfaces with enhanced ionic conductivity or electrochemical stability, achieving targeted interface property modulation through latent space manipulation. During the generation process, attention mechanism, integrated gradients, or SHapley Additive exPlanation (SHAP) values can highlight which compositional or structural motifs contribute most to the predicted target properties. These signals can be cross-checked with chemical motif detection tools such as Pymatgen (*63*) for coordination analysis and Spglib (*181*) for symmetry analysis. For example, based on the SHAP values, the Li content, vibrational amplitude, and Li^+^ interstitial defects were highlighted as critical factors affecting Li^+^ transport within SEs (*182*).

To bridge the persistent gap between computational predictions and experimental observations, a multimodal contrastive learning framework can be developed for SEI/CEI optimization, enhancing model transparency by making latent space embeddings more structured and chemically meaningful. This approach establishes a unified representation space through the synergistic integration of (i) extracting DFT-derived features, such as interfacial atomic configurations (e.g., Li_3_PS_4_/Li coordinates), electronic density of states, and ion migration barriers; (ii) deconvoluting solid-state nuclear magnetic resonance (NMR) chemical shift spectra into quantitative descriptors (peak positions reflecting Li^+^ coordination environments, linewidths indicating structural disorder) (*129*, *183*) based on operando characterization; and (iii) implementing convolutional neural networks for automated extraction of morphological features (GBs and crack propagation) and elemental distribution patterns from scanning transmission electron microscope images (*184*, *185*). By quantitatively correcting computational errors through such calibration, the framework effectively mitigates discrepancies with experimental observations, thereby improving the reliability of AI-guided design for both SEs and SEI/CEI.

By jointly embedding data from simulations and experiments into a shared latent space, contrastive learning frameworks can explicitly align representations of corresponding computational-experimental pairs while maximizing separation from mismatched pairs. This enables the model to learn systematic mapping functions that account for experimental artifacts, kinetic limitations, or measurement noise. Such cross-modal alignment improves the transferability of computational insights to real-world conditions. Moreover, from the latent correlations across modalities, AI can generate testable mechanistic hypotheses. For example, unified embeddings can reveal that certain SEI compositional motifs consistently correspond to low interfacial resistance across both simulated and experimental data or that specific microstructural features seen in tomography images align with computational predictions of ionic transport bottlenecks. This iterative, AI-guided loop between computation and experiment has the potential to accelerate not only materials discovery but also the fundamental understanding of interfacial phenomena in SSBs.

##### Direction 3: Building an intelligent ecosystem integrating AI, computations, and experiments

The absence of an intelligent ecosystem for SSBs stems primarily from the disjointed nature of computational materials modeling, experimental research, and AI. Computational tools and experimental methodologies vary across research teams and laboratories, often lacking standardization, which hinders data sharing, integration, and reusability. Addressing this issue requires the development of an open, scalable intelligent platform that seamlessly integrates AI algorithms, physical simulation tools [such as DFT, MD, and phase-field models (*174*)], and experimental materials databases. Such a platform would enable unified data management, model training, experimental analysis, and predictive modeling, streamlining research workflows. Despite the abundance of experimental results on SEs and SEI/CEI layers scattered across decades of publications, much of this information remains underused due to its unstructured and heterogeneous nature. AI methods, particularly natural language processing and data mining techniques, offer powerful tools to automatically extract and standardize such information from scientific literature. For instance, transformer-based models [e.g., MatSciBERT (*186*), MaterialsBERT (*187*), and BatteryBERT (*188*)] can be fine-tuned to recognize material names, compositions, synthesis conditions, interfacial observations (e.g., LiF-rich SEI and dendrite suppression), and performance metrics across thousands of papers. By integrating these extracted data into structured databases or knowledge graphs, AI can enable cross-study comparisons, trend identification, and gap analysis, which are difficult to achieve via manual review. Moreover, the SSB ecosystem must establish standardized materials data formats and data-sharing protocols, allowing for seamless access and fostering a truly intelligent and collaborative research network.

Beyond data and computational integration, experimental automation and AI-driven adaptive optimization serve as crucial pillars for an intelligent research ecosystem. In catalysis and drug discovery, automated laboratories—such as the self-driving experimental systems pioneered at MIT—use robotic platforms to autonomously synthesize, characterize, and evaluate materials while using AI to analyze experimental data in real time and dynamically adjust experimental parameters (*189*). However, this paradigm has yet to be widely adopted in the SSB field, largely due to the inherent complexity of SE synthesis and SEI/CEI interfacial characterization. Furthermore, the fabrication of SSBs involves multiple intricate processes, including sintering, thin-film deposition, and mechanical pressing, each of which critically influences final performance, complicating AI-driven optimization. To overcome these challenges, an adaptive experimental framework leveraging techniques such as multifidelity Bayesian optimization (MFBO) (*190*), flow-driven data intensification (FDI) (*191*), and reinforcement learning [e.g., policy gradient network (PGN) and deep Q-network (DQN) (*192*)] can be integrated with robotic laboratories to efficiently explore optimal synthesis conditions across multiple parameters (e.g., temperature, pressure, and doping concentrations). For instance, use MFBO to integrate low-cost DFT screening with high-fidelity experiment data to search the SE structures with low interfacial resistance, and dynamically prioritize experiments on SEI/CEI compositions based on FDI. Then, PGN/DQN can learn adaptive synthesis protocols to stabilize SEI/CEI under mechanical and electrochemical stress. By designing each iteration to target underexplored regions in composition-processing-property space, experimental results can be directly used to refine AI models, improving their predictive accuracy. Integrating uncertainty quantification can further guide experiments toward the most informative data points, accelerating the SE and interface engineering.

Establishing a truly intelligent SSB ecosystem also necessitates dismantling disciplinary silos to foster interdisciplinary collaboration. Research in this field spans materials science, computational chemistry, condensed matter physics, AI, and engineering, yet integration across these domains remains fragmented. Computational materials scientists often focus on the underlying physical mechanisms of DFT and MD simulations, whereas experimentalists prioritize synthesis techniques and performance characterization, leading to inefficiencies in the convergence of theory and experiment. To bridge this gap, future research must embrace an interdisciplinary paradigm through the establishment of collaborative research hubs, the co-development of AI-driven material design tools [e.g., self-driving laboratories (SDLs) (*193*, *194*) and AI agents (e.g., stable noisy optimization by branch and gradient decent Gaussian process) (*195*)], and the sharing of multiscale experimental and computational datasets. Government agencies and industry stakeholders should provide financial and infrastructural support to foster interdisciplinary talent, promote academia-industry partnerships, and develop AI-powered platforms tailored for scalable SSB design. Beyond accelerating fundamental research, these efforts should prioritize cost-effective materials, large-scale manufacturing compatibility, and rigorous lifetime and safety assessments. By integrating researchers, engineers, and manufacturers within a unified digital ecosystem, AI-driven innovations can bridge the gap between laboratory discoveries and industrial adoption, expediting the commercialization of SSBs and advancing sustainable energy solutions.

## DISCUSSION

In this review, we offer an in-depth exploration and discussion of the AI-driven applications for the SE design and interfacial engineering, based on the recent literature, including the structure-property relationship-driven materials screening pipelines, MLFFs for MD simulations, and cross-scale modeling for unveiling complex physicochemical mechanisms. These advancements have laid a crucial technical foundation and provided valuable research insights for applying cutting-edge AI approaches in the development of SSBs. Then, we further conduct an in-depth investigation into the unresolved key challenges in modeling complex multiscale structure-property relationships, developing multimodal models embedded with physical constraints, and speeding up the efficient and green development of SSBs in the era of AI. We also propose highly feasible potential solutions, including coupling multiscale modeling via MLFFs and bidirectional optimization, developing multimodal models embedded with physical constraints, and building an intelligent ecosystem that seamlessly combines AI, computations, and experiments.

In the near term (0 to 2 years), regular academic and industrial laboratories can take important preparatory steps to pave the way for AI integration without relying on fully autonomous platforms. These include developing standardized experimental protocols for synthesis, characterization, and reporting to ensure data quality and reproducibility, which are essential prerequisites for any AI modeling. In parallel, large-scale computational datasets can be extended using MLFFs to simulate behavior under diverse operating conditions. For SEs and SEI/CEI studies, a benchmark experimental dataset should be established that includes controlled synthesis procedures, environmental conditions, uniform measurement techniques, and complete data documentation. Such efforts create the necessary data infrastructure for future AI applications.

In the mid-term (2 to 5 years), individual laboratories can begin to collaborate with AI researchers to apply active learning, design of experiments, or data-driven modeling to guide experimental campaigns. By systematically varying synthesis parameters, doping concentrations, and interfacial modifications, researchers can explore broader structure-property relationships while building high-value datasets. Data fusion techniques can help combine information from different experimental platforms while accounting for measurement inconsistencies, enabling richer model training. Even in the absence of automation, AI can be used to prioritize experiments, uncover hidden trends, and identify promising composition ranges.

In the long term (5+ years), the community can move toward autonomous, AI-guided experimentation frameworks, where ML models not only predict properties but also recommend optimal synthesis routes or interfacial treatments to achieve desired performance. While full SDL may take time to be widely adopted, establishing community-wide, standardized materials databases with unified formats and shared ontologies will allow AI models trained in one laboratory to generalize across institutions. This will be a key step toward accelerating commercialization of AI-designed SEs and interfaces, bridging the gap between academic research and industrial application. Given the rapid progress in materials science, energy science, and AI technologies, we anticipate that in the near future, AI will achieve substantial breakthroughs in SSB research and application.
