# Supplementary material for: Toward AI ecosystems for electrolyte and interface engineering in solid-state batteries
Source: Sci Adv. 2025 Nov 26;11(48):eaea0638. doi: 10.1126/sciadv.aea0638 (PMC12652334; doi:10.1126/sciadv.aea0638)
Supplement: Supplementary file 1 — Tables S1 to S5 [file sciadv.aea0638_sm.pdf]

Science Advances
AAAS

Supplementary Materials for

Toward AI ecosystems for electrolyte and interface engineering in solid-state batteries

Zhilong Wang *et al.*

Corresponding author: Fengqi You, fengqi.you@cornell.edu

Sci. Adv. **11**, eaea0638 (2025)
DOI: 10.1126/sciadv.aea0638

This PDF file includes:

Tables S1 to S5

Table S1.

Previous reviews on SE and interfacial design for high-performance SSBs. These reviews focus on the research progress of SEs and/or interfaces in SSBs through experiments, computations, and/or AI techniques (screening pipeline, MLFF, and generative models). Currently relatively few comprehensive discussions on AI-driven SE and interface design, means that computational and AI-based studies in SSB research are still in the preliminary stages.

**Previous review**	**Main finding**	**Main scope**	**AI coverage**	**Source**
**1**	The bottlenecks and the related solutions regarding organic/inorganic composite electrolyte, lithium-metal anode, and high-voltage cathode were focused on, and compatible interface engineering was presented.	SE and interface	/	(*41*)
**2**	An overview of the development and characteristics of SEs was provided, followed by analysis of ion transport in the bulk and at interfaces based on different single-valent (Li^+, Na^+, K^+) and multivalent (Mg^{2+}, Zn^{2+}, Ca^{2+}, Al^{3+}) cation carriers of contemporary interest.	SE and interface	Screening pipeline	(*42*)
**3**	The experimental findings for various classes of SEs were summarized and related to computational predictions, with the aim of providing a deeper understanding of the interfacial reactions and insight for the future design and engineering of interfaces in SSBs.	SE and interface	/	(*17*)
**4**	The recent development of garnet-type electrolytes with discussions of experimental studies and theoretical results were reviewed.	SE	Screening pipeline	(*43*)
**5**	The development of halide battery chemistry, the preparation, modification, and properties of halogen-containing SEs were focused on.	SE	/	(*50*)
**6**	The key challenges that still involve the need for fast-conducting SEs were reviewed to provide sufficient transport in composite cathodes. It was proposed that the high-performance anodes together with protection concepts are paramount to establish dense high-energy SSBs.	SE	/	(*6*)
**7**	An overview of different numerical methods used in SE research was provided. The current state of knowledge in numerical auxiliary approaches, with a particular focus on AI-enabled methods, for the understanding of multiphysics-couplings of SEs at various spatial and time scales was discussed.	SE	Screening pipeline and MLFF	(*197*)
**8**	A detailed classification of the various "windows" of SEs and a comprehensive understanding of the associated interfacial stability of SEs in full battery application was provided.	SE and interface	/	(*198*)
**9**	An introduction to the background of SEs, including their explicit definition, comprehensive classification, intrinsic physical/chemical properties, underlying mechanisms governing their conductivity, challenges, and future developments was provided. An in-depth explanation of the AI methodology was also elucidated.	SE	Screening pipeline	(*40*)
**10**	The progress in using atomistic modelling and AI techniques to gain valuable insights into inorganic crystalline SEs for Li-based and Na-based batteries was discussed.	SE	MLFF	(*199*)

Table S2.

Commonly used toolkits for constructing MLFFs. It should be noted that the information of applicable systems, system size, and data requirements is summarized by the previous usage.

Model	Applicable systems	System size	Data requirements	Hardware requirements	Source
**DeepMD**	Metals, oxides, semiconductors, electrolytes, aqueous systems	10^3–10^5 atoms	High, ~10^4 samples	GPU required, TensorFlow-based	(*161*)
**M3GNet**	Inorganic materials, crystals, alloys	Up to ~10^4 atoms	Medium, 10^3–10^4 samples	GPU recommended (PyTorch-based)	(*165*)
**CHGNet**	Charged systems, ionic materials, solid-state electrolytes	~10^3 atoms	Medium, 10^3–10^4 samples	GPU recommended (PyTorch-based)	(*200*)
**GAP**	Covalent, metallic, and some ionic materials	Up to ~10^3 atoms	Very high, ~10^5 samples	High-performance CPU preferred, OpenMP parallelism	(*201*)
**SchNet**	Molecular systems, small organic/inorganic crystals	Up to ~10^3 atoms	High, ~10^4 samples	GPU recommended (PyTorch-based)	(*202*)
**NequlP**	Molecules, liquids, solid-state materials	10^3–10^4 atoms	High, ~10^4 samples	High-end GPU (PyTorch-based)	(*203*)
**ACE**	Metallic alloys, complex oxides, semiconductors	Up to ~10^3 atoms	High, ~10^5 samples	High-performance CPU	(*204*)
**ANI**	Organic molecules, biomolecules	10^2–10^3 atoms	Medium, 10^3–10^4 samples	GPU recommended	(*205*)
**PINN**	Covalent materials, semiconductors	Up to ~10^4 atoms	Medium, 10^3–10^4 samples	GPU recommended	(*206*)

Table S3.

Representative generative models for materials discovery. RMSD (Å): root mean squared displacement between generated and DFT-relaxed structures. Perov-5: 18,928 perovskite materials; Carbon-24: 10,153 materials made up of carbon atoms; MP-20: 45,231 inorganic materials; Alex-MP-20: 607,683 stable structures with up to 20 atoms.

**Model**	**Architecture**	**Materials System**	**Generation performance**	**Code Link**	**Source**
**G-SchNet**	GNN	MP-20	RMSD = 1.347	https://github.com/atomistic-machine-learning/G-SchNet	(*75*)
**PGCGM**	GAN	2000 inorganic compounds	93.45%	https://github.com/MilesZhao/PGCGM	(*77*)
**CDVAE**	VAE	Perov-5, Carbon-24, MP-20	Recall = 99.4%, 99.8%, 99.1%	https://github.com/txie-93/cdvae	(*78*)
**UniMat**	Diffusion model	Perov-5, Carbon-24, MP-20	Recall = 99.2%, 100.0%, 99.8%	https://unified-materials.github.io/unimat/	(*80*)
**SMLD**	GNN	MP-20	Recall = 96.5%	https://github.com/yang-song/score_sde	(*81*)
**SLICES**	RNN	MP-20	Structural validity = 99.72%	https://github.com/xiaohang007/SLICES	(*82*)
**MatterGen**	Diffusion model	Alex-MP-20	RMSD = 0.021	https://github.com/microsoft/mattergen	(*31*)

Table S4.

Summary of predictive models for discovering SEs with high ionic conductivity. The inputs, outputs (targets), AI models, model types, and model performances are listed.

Input	Output	Model	Type	Performance	Source
Structure, composition	Fast-ion conductor labels	LR	Classification	Accuracy=90%	(*88*)
Structure, composition, site	Fast-ion conductor labels	XGBoost	Classification	Accuracy=90.4%	(*67*)
Structure	Ionic conductivity	GCN	Regression	MAE=1.558 (at $\log_{10}$ level)	(*90*)
Structure	Activation energy	CGCNN	Regression	MAE=0.19 eV	(*92*)
XRD	Fast-ion conductor labels	VGG-11	Classification	Accuracy=92%	(*96*)
SEM microstructure	Fast-ion conductor labels	ResNet	Classification	Accuracy=95.9%	(*94*)
Structure, composition	Fast-ion conductor labels	RF	Classification	Accuracy=88.7%	(*98*)
Structure, composition, transportation, distribution	Fast-ion conductor labels	Linear discriminant analysis	Classification	Accuracy=81%	(*97*)
Composition	Ionic conductivity	Extremely randomized trees	Regression	MAE=0.603 (at $\log_{10}$ level)	(*98*)
Composition	Ionic conductivity	DopNet with Li attention mechanism	Regression	Coefficient of determination=0.84	(*99*)

Table S5.

Summary of predictive models for SEI/CEI optimization. The inputs, outputs (targets), AI models, model types, and model performances are listed.

Input	Output	Model	Type	Performance	Source
Anode-SE interface	Kinetic stability label	Logistic regression	Classification	Accuracy=78% (stable model), accuracy=74% (reactive mode)	(*133*, *134*)
Anode-SE interface	XPS binding energy	XGBoost	Regression	MAE=0.03 eV, RMSE=0.04 eV	(*135*)
SEI composition	Ground-state phase K_{crit}	Random forest	Regression	RMSE=2.59 GPa	(*136*)
SEI composition	Decomposition energy and critical modulus	Decision tree	Regression	RMSE=0.063 eV/atom (decomposition energy), RMSE=0.91 GPa (critical modulus)	(*137*)
Coating structure for CEI	Energy and forces	MLFF with active learning	Regression	MAE=2.96 meV/atom (energy), MAE=63.62 meV/Å (force)	(*138*)
Cathode-SE interface	Energy and force	MLFF	Regression	RMSE=2.5 meV/atom(energy), RMSE=109.7 meV/Å (force)	(*139*)
Cathode interface	Energetic favorable Interfacial atomic positions	PSO with CALYPSO	Optimization	The generated structure is more than 2.7 eV lower in energy than the randomly obtained structure.	(*140*)
Cathode interface	Dynamic evolution of CEI	MLFF	Regression	RMSE=~20meV/atom (energy), RMSE = ~0.27 eV/Å (force)	(*55*)
Cathode interface	Energy and interface structure	MLFF	Regression	Coefficient of determination=0.95	(*86*)
